# Assessment of drag measurement techniques in a shock tunnel

**DOI:** 10.1371/journal.pone.0270743

**Published:** 2022-07-08

**Authors:** Keunyeong Kim, Byungkook Jang, Sanghoon Lee, Gisu Park

**Affiliations:** Department of Aerospace Engineering, Korea Advanced Institute of Science and Technology, Daejeon, Republic of Korea; University of Genova, ITALY

## Abstract

Three force measurement techniques in a shock tunnel, the free-flight, movable-support force balance, and stress-wave force balance techniques were employed, and each technique’s characteristics were assessed. For each force measurement technique, the system setup, data processing method, measurement uncertainties, and applied range of the test model size-flow establishment time were described in detail and compared. For a comparison and discussion, the drag coefficients of a circular pointed cone model with a semi-angle of 18.4° at a nominal freestream Mach number of 6 were measured. As a result, three force measurement techniques yield similar drag coefficients. However, the measurement uncertainties were increased in the order of the free-flight, the stress-wave force balance, and the movable-support force balance techniques. The main causes of the measurement uncertainties were the corner detection uncertainties for the free-flight techniques, and the propagation of the internal or external vibrations for the movable-support and stress-wave force balance techniques. To estimate the appropriate range of the test model size and flow establishment time for each technique’s application, the force measurement systems of the present work and the available literature were compared. As a result of comparative discussion, force measurement environments that can be advantageous for each technique are suggested.

## Introduction

For a hypersonic vehicle, a strong shock wave is generated in front of the surface. Due to the strong shock wave, the flow pressure rises both significantly and suddenly, and this causes a dramatic increase in the pressure drag of the vehicle. The dramatic increase of the pressure drag induces the larger fuel consumption rate for the propulsion system [1] and requires the geometry modification of the vehicle for drag reduction [2]. Since the pressure drag can attain above the 50% of the total drag, the design of a hypersonic vehicle is dominated by the pressure drag [3, 4]. Therefore, it is important to predict the drag of the vehicle in hypersonic flight conditions. Given that the real-flight tests require a enormous budget and are very limited, ground test facilities have been widely used to measure the drag for the scaled test model in a specific flight condition.

Impulse facilities are one of the ground test facilities that can simulate a wide range of high-pressure and high-temperature supersonic flow conditions with real-gas effects for drag measurements. In recent years, great efforts have been made to develop force measurement techniques with a high accuracy and fast responses for impulse facilities to overcome the limitations in impulse facilities of the short steady test time [5] and non-stabilized structural vibrations inside the model-support system.

Depending on the measuring properties for determining the drag, the drag measurement techniques can be primarily categorized as acceleration measurement-based techniques [6, 7] and strain gauge-based techniques [8, 9]. The representative techniques of acceleration measurement-based techniques are the free-flight technique (FFT) and the movable-support force balance technique (MST), whereas the stress-wave force balance technique (SWT) represents strain gauge-based techniques.

The acceleration measurement-based techniques measure the acceleration of the test model to determine the aerodynamic forces using Newton’s second law of motion. In the acceleration measurement-based techniques, the model support is removed or weakened in order to obtain the acceleration of the test model. For the FFT, detachable supports, which allow the test model to move freely during the test time, are adopted to measure the acceleration of the lightweight test model [6, 10, 11]. By using the detachable supports, the aerodynamic forces can be measured, minimizing flow disturbance from the model supports. However, the maximum mass of the test model is limited because detachable supports can only hold a relatively light test model. In addition, the flow establishment time around the test model should be as short as possible in order to avoid any disturbances to the aerodynamic forces by the motion of the test model during the flow establishment time.

The MST uses the movable-support type model-supporting mechanism to hold the test model and measures the test model’s acceleration [7, 12, 13]. The movable supports, which usually consist of rubber bushes or ball bearings, hold the test model flexibly and give the test model quasi-free body motion only for small displacement with a short duration. Compared to the detachable supports in the FFT, the movable supports are adopted in the MST so that the drag measurements of relatively large and heavy test models with high repeatability can be possible. However, the maximum test model mass in the MST is also limited to obtain the significant acceleration of the test model during a short test time. Also, because the test model of the MST is usually held by the movable support using sting, the minimum test model mass, including the sting, is limited.

The SWT, one of the most widely used strain gauge-based techniques, determines the applied aerodynamic forces from the deformations of the model-support system, i.e., strain [8, 14, 15]. In the strain gauge-based techniques, the non-stabilized structural vibrations in the strain signal of the system should be minimized or calibrated for short duration measurements. For the SWT, the non-stabilized structural vibration in the strain signal is regarded as a system’s dynamic response, not as a disturbance, and the aerodynamic force is recovered from the vibrating strain signal using an inverse technique based on the system’s dynamic response function. In order to determine the dynamic response function of the system, a dynamic calibration should be performed for the force measurement system. Unlike the acceleration measurement-based techniques of the FFT and the MST, the SWT can be used for the drag measurements of large and heavy models because the model-supporting mechanism can hold the test model rigidly. In addition, because the test model can be held rigidly and the test model motions are minimized during the flow establishment time, the SWT can be used for the test conditions with longer flow establishment times. Because the accuracy of the force measurements using the SWT is strongly dependent on the compensation and calibration of the non-stabilized structural vibrations, dynamic calibrations with high accuracy are required for the SWT and the system’s dynamic response function should be accurately determined. In addition to the SWT, the stiff force balance [16–18] and the acceleration-compensated strain gauge [19–21] systems have also been developed as the strain-gauge-based techniques.

As mentioned, due to the different model-supporting mechanisms, the FFT, MST, and SWT have different system characteristics for the system setup, data processing methods, and an appropriate range of model mass and flow establishment time for the technique’s application. To accurately measure the drag in impulse facilities, the technique should be appropriately selected for the specific test environments. This can be achieved by performing the comparative assessment of the techniques under the specific test environments, based on the rigorous understanding of each technique’s characteristics and experimental application. Although comparative studies between two of three force measurement techniques have been conducted [22–25], the direct comparisons and discussions of all three techniques regarding the system setup’s key factors and the appropriate range of test model sizes for the technique’s application are insufficient.

In the present work, the comparative assessment of three drag measurement techniques of the FFT, MST, and SWT was performed using a shock tunnel. The drag coefficients were measured with the three drag measurement techniques under Mach 6 test flow condition. Each technique’s system setup, data processing method, measurement uncertainties, and appropriate ranges for the test model size and flow establishment time for the technique’s application were described in detail. The uncertainty analysis for each technique was performed. In addition, the force measurement system setup from the present work and the available literature are compared, and the applied range of the model size and flow establishment time were compared to estimate the appropriate range of the test environments for each technique. The experimental methods and results could provide guidelines for the drag measurements using impulse facilities.

## Experimental details

### Test facility

**Fig 1:**
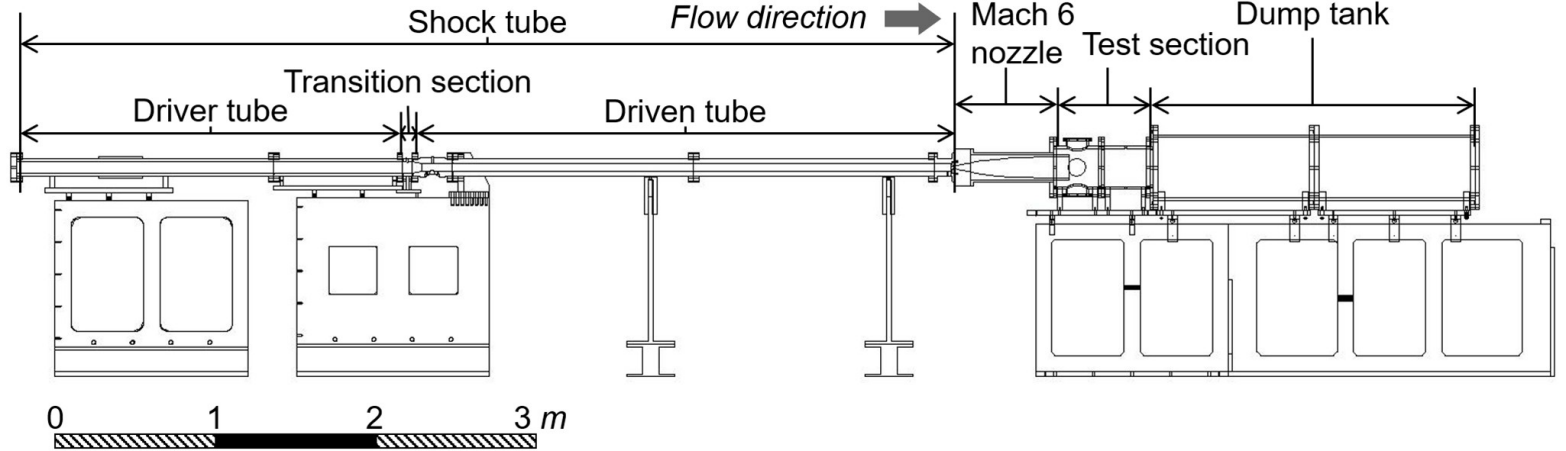
Schematic of K1 shock tunnel.

Fig 1 represents the schematic of the K1 shock tunnel test facility for the drag measurements in this study. The K1 shock tunnel was a conventional pressure-driven shock tunnel [26, 27] which consisted of a shock tube, a nozzle, a test section, and a dump tank. The shock tube was composed of three axially symmetric parts: a driver tube (length of 2.4 *m*), a transition section (0.06 *m*), and a driven tube (3.6 *m*). The inner diameters of each part were 68, 68, and 47.5 *mm*, respectively. At the driven tube’s end wall, a Mach 6 contoured converging-diverging nozzle was installed using an adapter. The nozzle had an exit diameter of 128 *mm* with an exit-to-throat area ratio of 60.6. The test section was directly attached to the nozzle adapter. Though not shown in the figure, in total, three optical windows were then located in the test section. Two optical windows were located sidewise with opposite positions, and another optical window was located top of the test section. Three optical windows were used for the FFT; two sidewise windows to capture the motion of the test model in the axial and pitch directions, and the motion of the test model in the axial and yaw directions were captured through the top window.

The operation of the test facility was started with the incident shock wave generation from the pressure difference across the driver tube, the transition section, and the driven tube. The test gas was primarily compressed due to the propagation of the incident shock wave and was compressed secondly by the reflected shock wave, which was generated when the incident shock wave is reflected at the driven tube end wall. The shock tube reservoir flow behind the reflected shock wave was expanded and accelerated through the nozzle and reaches the test section. The static pressure behind the incident shock wave *P*_2_ was measured by the two flush-mounted piezoelectric pressure transducers (Model: 111A23, Piezotronics) installed at the sidewall of the shock tube with a constant interval of 68.5 *mm*. Both the flow pressure near the end wall (regarded as the shock tube reservoir total pressure *P*_0_) and the nozzle-exit pitot pressure *P*_*pt*_ were measured using a flush-mounted pressure transducer (Model: 111A26, Piezotronics). It should be noted that the near-end wall pressure is measured 45.5 *mm* upstream of the sidewall of the shock tube end wall in order to avoid the pressure transducer damage by the direct impact of the incident shock wave.

In this work, for stable signal triggering, the measurement systems in the test section were synchronized with the near-end wall pressure transducer and started measuring before the test flow arrived at the test model. To set the time when the test flow reached the test model to the zero-point, all measurement results in the present work were shifted in the time of -0.43 *ms* and plotted. The pitot pressure at the nozzle exit and the drag coefficient of the test model were independently measured.

### Flow condition

For the present test flow condition, tailoring technique [28] was applied for the driver gas to extend the test time of the test facility. In the previous work [29], the tailoring technique was performed by the driver gas tailoring. The driver gas tailoring technique adjusts the composition of the driver gas to eliminate the flow disturbances caused by the interaction between the reflected shock wave and the contact surface (the interface of the driver and the test gas); and, the test time can be extended. As a result of the previous work, the test time was extended from 0.7 to 3.2 *ms* [29]. In this work, the tailored test condition of the previous work was used, and the tailored driver gas composition was 97% high purity helium mixed with 3% nitrogen by volume. The transition section was filled with 100% helium. The driven tube was filled with dry air. The pressure conditions of the driver tube, the transition section, and the driven tube were 3.2, 1.7, and 0.04 *MPa*, respectively.

Table 1 summarizes the steady-state flow properties at the shock tube reservoir and the nozzle exit with the tailored condition. In the table, the symbol “±” indicates a 95% confidence level for each of the properties based on three-multiple shots, which indicates the repeatability of each property measurement. *v* is speed, *P* is flow pressure, *T* is flow temperature, *ρ* is density, *h* is flow enthalpy, and *M* is Mach number. Subscript *S* indicates the incident shock wave, 0 indicates total (or stagnation) property, and ∞ indicates freestream property. In the “Experiment” column, the speed of the shock wave *v*_*S*_ was calculated via dividing the distance between the two-sidewall static pressure transducers by the difference of the arrival timing of the incident shock wave at the two pressure transducers. The nozzle-exit Mach number *M*_∞_ was determined from the shock wave standoff distance measurements around a hemisphere model using a Z-type shadowgraph technique. According to the equation proposed by Serbin [30], the density ratio across the shock wave can be calculated from the shock wave standoff distance-to-hemisphere diameter ratio. Then, the freestream Mach number can be obtained using the calculated density ratio with the normal shock wave relation. The details regarding the Mach number measurements based on the shadowgraph images are presented in Ref. [31]. The flow properties at the shock tube reservoir in the “Calculation” column were calculated by using a gas dynamics calculator [32], which uses the Rankine-Hugoniot relations with the Mach number of the incident shock wave as the input [33]. For the calculation, it was assumed that the flow is isentropic, and that real-gas effects are negligible. Considering that the total temperature *T*_0_ for the present test flow condition was lower than 2,000 *K* where chemical reactions hardly occur, these assumptions were reasonable. Then, the flow properties at the nozzle exit was determined from the calculated flow properties at the reservoir using isentropic expansion relations. The nozzle-exit pitot pressure was calculated from the flow properties at the nozzle exit using the well-known Rayleigh pitot relation [33].

**Table 1:** Test flow condition for drag measurements.

Location	Property	Experiment	Calculation
Shock tube reservoir	*v*_*S*_ [*m*/*s*]	1130 ±13.6	1130
	*P*_0_ [*MPa*]	2.81 ±0.08	2.73
	*T*_0_ [*K*]	-	1640
	*ρ*_0_ [kg/m3]	-	5.54
	*h*_0_ [*MJ*/*kg*]	-	1.57
Nozzle exit	*M*_∞_ [-]	5.98 ±0.28	5.9
	*P*_∞_ [*kPa*]	-	1.90
	*T*_∞_ [*K*]	-	212
	*ρ*_∞_ [kg/m3]		0.03
	*P*_*pt*_ [*kPa*]	84.4 ±2.23	86.1

**Fig 2:**
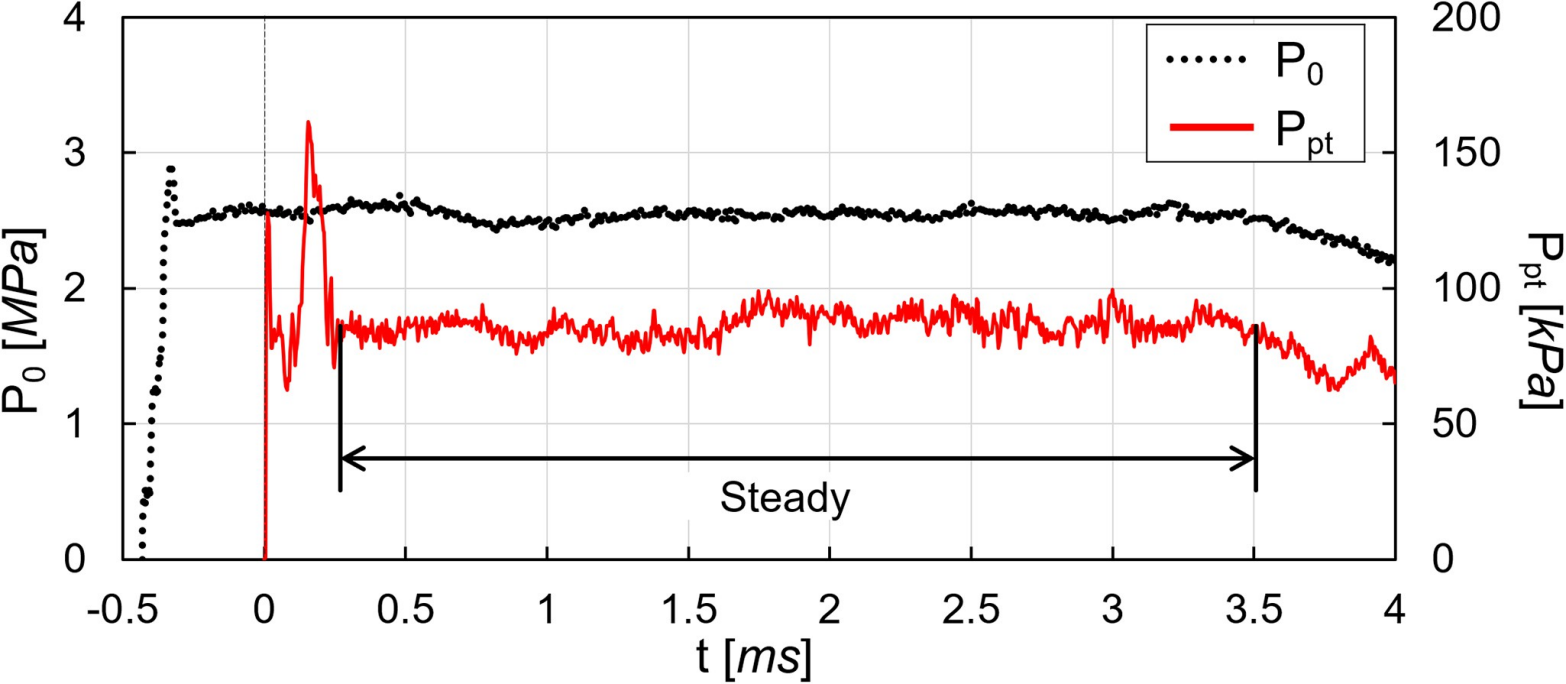
Typical measured pressure histories in K1 shock tunnel experiment.

Fig 2 shows the representative time histories of the near-end wall pressure *P*_0_ and the nozzle-exit pitot pressure *P*_*pt*_ measurements. The near-end wall pressure is represented at the right vertical axis with a dotted black line, while the nozzle-exit pitot pressure is represented at the left vertical axis with a solid red line. For the comparison, the near-end wall pressure and the nozzle-exit pitot pressure are denoted with *MPa* and *kPa*, receptively. The horizontal axis represents the time *t* with milliseconds. The rise of the nozzle-exit pitot pressure is 0.43 *ms* slower than the rise of the near-end wall pressure because the nozzle-exit pitot pressure measurement was started after the expansion of the shock tube reservoir flow through the nozzle. As mentioned, the rise timing of the nozzle-exit pitot pressure histories was set as zero for the time in the present work; therefore, the rise of the near-end wall pressure was started at -0.43 *ms*. The rapid rise of the nozzle-exit pitot pressure from 0.1 to 0.25 *ms* was possibly originated from the supersonic nozzle staring process. For the nozzle-exit pitot pressure *P*_*pt*_, the flow maintained the steady-state for 3.2 *ms* (from 0.3 to 3.5 *ms* in the figure) after the flow development.

## Drag measurement technique setup

In the present work, three different techniques were used to measure the drag coefficient in the shock tunnel. The drag coefficient measurements were performed in the identical flow condition in Table 1. For the geometry of the test model, the identical shape of a circular pointed cone with a semi-angle of 18.4°, and a base diameter and length of 24 and 36 *mm*, respectively, was used for the three different techniques. However, the mass and the material for the test model differed depending on the technique. Based on the principle of drag coefficient acquisition, different model-supporting mechanisms and system setups were required. In this section, the model-supporting mechanism and the system setup of each technique are described in detail. The drag coefficient acquisition procedure using each technique are described in ‘**Data processing**’.

### Free-flight technique setup

The FFT measures the acceleration of the free-flying test model without supports and obtains the drag of the test model using Newton’s second law of motion and the measured acceleration of the test model. Because of the simplicity of the system setup and the negligible support effects, the FFT is the most fundamental method for force measurements and has the lowest disturbance effect from the support to the flow field and the measurement system.

**Fig 3:**
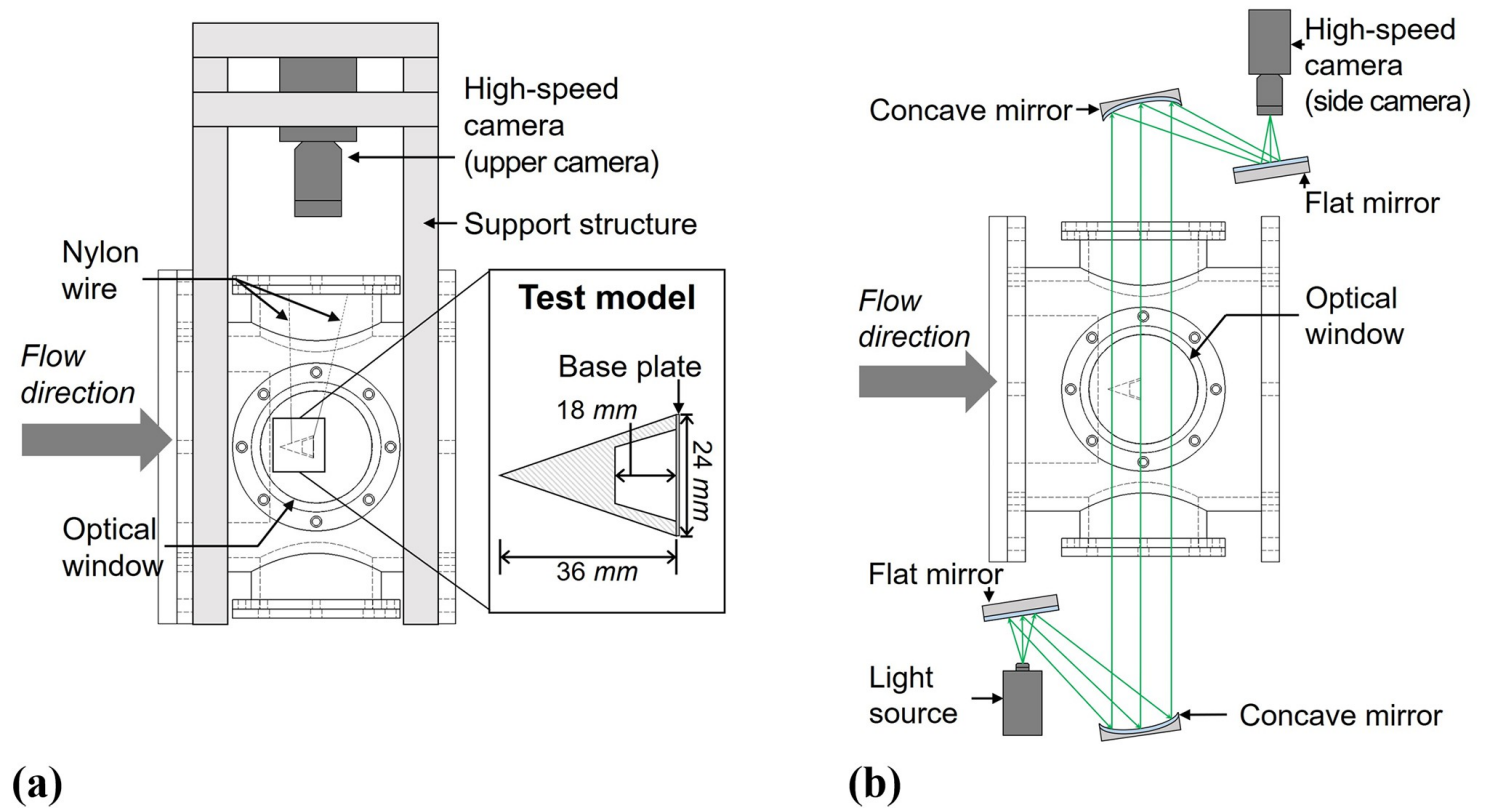
Schematic of the FFT setup. (a) Side view, (b) Top view.

Fig 3 shows a schematic of the FFT setup. In the present work, the test model was allowed for free-flight during the test time using detachable wires, and the accelerations of the free-flying model were obtained by the optical tracking method. As can be seen in Fig 3(a), the test model was held by the test section using two 0.4 *mm*-nylon wires with glue. The wires were attached near the tip and the base of the model, and the other ends of the wires were attached at the upper optical window. The length of the wire was adjusted in order to control the initial posture of the test model. When the test flow reached the test model, the wires were detached from the surface of the test model, and the model began free flight. The test model was made of acrylic, which is light and has stable mechanical properties. To ensure longitudinal stability, the test model had a tapered cavity in order to reduce the distance between the center-of-mass and the center-of-pressure, as shown in Fig 3(a). A 0.3-*mm* thick polycarbonate sheet was attached to the model base to reduce the flow disturbances caused by the tapered cavity. The resulting weight of the test model was approximately 2.59 *g*.

For the FFT, the test model is in a free-flight; therefore, unexpected motions other than axial motions, e.g., yawing or pitching motions, can occur. Thus, in this work, two different optical techniques were used to check the pure axial motion of the test model, and only the drag with the pure axial motion of the free-flying test model was obtained. The upper camera in Fig 3(a) was used to capture the yaw and the axial motion images, while the side camera with the Z-type shadowgraph setup [10, 31, 34] in Fig 3(b) captured the model’s pitch and axial motion images. The upper camera was fixed to the support structure and directly captured the images of the test model with a frame rate of 10,000 *fps* and a resolution of 1,280×480 *pixels*. The Z-type shadowgraph setup consisted of a high-powered light emitting diode (LED), two concave mirrors with a focal length of 1.5 *m*, two flat mirrors, and a high-speed camera with a frame rate of 40,000 *fps* and a resolution of 1,280×120 *pixels*. The Harris corner detector built-in function of MATLAB was employed to accurately detect the three vertexes of the test model in each frame [35]. When using the Harris corner detector, the Z-type shadowgraph images, which had high contrasts, were used to achieve the high accuracy of the detection results. Then, the displacements of the test model could be determined from the detected trajectories of the free-flying model. The acceleration of the test model could be obtained from the second derivative of the curve-fit model for the test model displacements.

### Movable-support force balance technique setup

The MST directly measures the acceleration of the test model using movable support and accelerometers in order to obtain the drag. The movable support is a model-supporting mechanism that weakly holds the test model and allows for quasi-free body motion of the test model during a test time. The representative movable supports are rubber bushes with an adequate stiffness [7, 13, 36], steel rollers [12], and steel wires [37].

**Fig 4:**
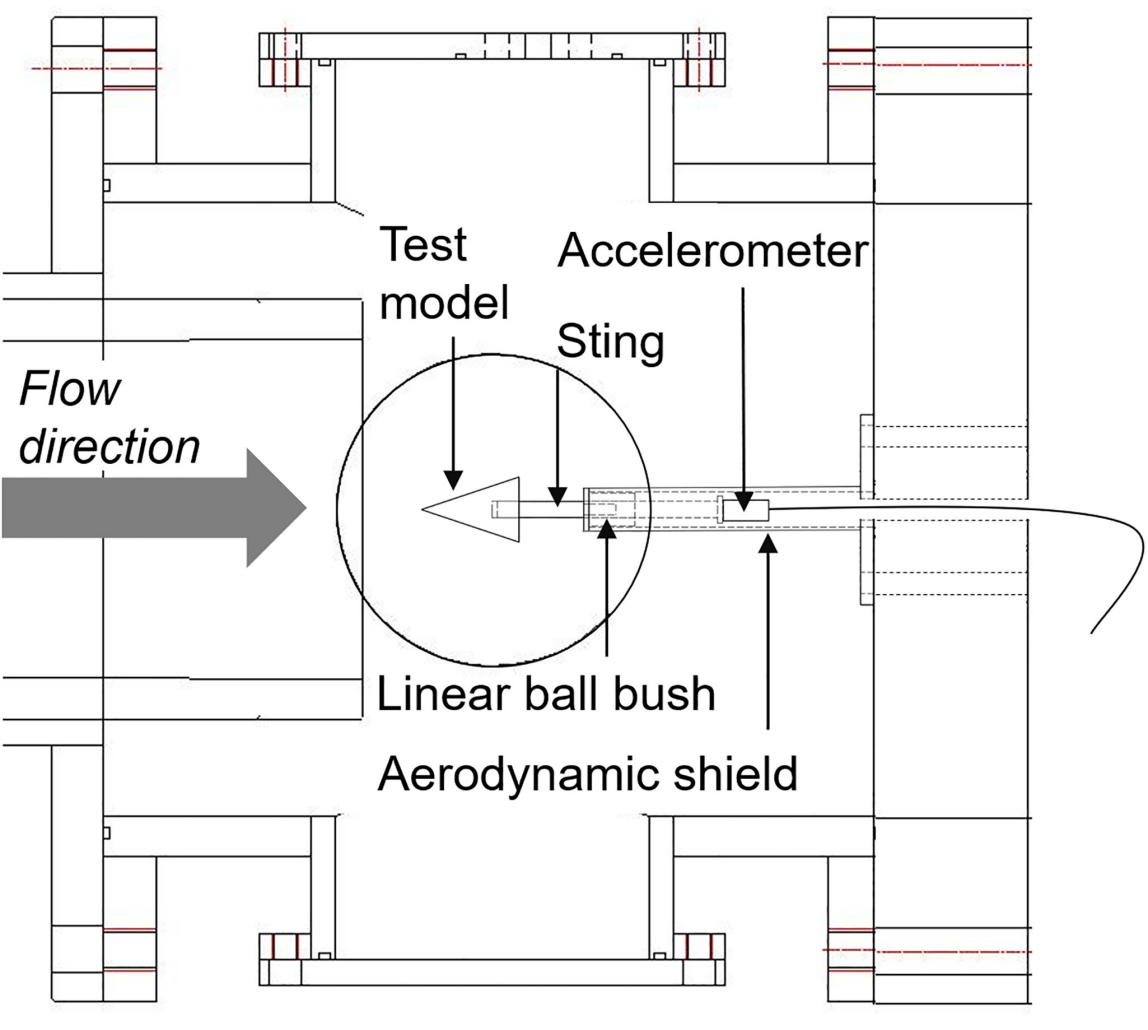
Schematic of the MST setup.

Fig 4 shows a schematic of the MST setup in the present work. The MST consisted of a test model, a sting, a linear ball bush, a aerodynamic shield, and an accelerometer. A commercial linear ball bush (Model: LM6UU, THK) was used to simulate the free-body motion of the test model in the axial direction and reduced the complexity of the system. Each part of the MST, except for the linear ball bush, was manufactured using an aluminum alloy Al-6061. The test model was fixed at the sting head, while the piezoelectric accelerometer (Model: 353B16, Piezotronics) was fixed at the sting tail in the axial direction. The length and diameter of the sting were 85 and 6 *mm*, respectively. The sting freely moved along the axial direction of the test model through the ball bush, with an external diameter of 20 *mm* and an inner diameter of 6 *mm*. In order to fix the linear ball bush and protect the accelerometer from the test flow, an aerodynamic shield having a diameter smaller than the test model was applied. The accelerometer was synchronized with the pressure transducer at the end wall of the shock tube, and the acceleration signal was recorded through an oscilloscope outside of the test section. The accelerometer signal, i.e., acceleration, was recorded for 5 *ms* at a sampling rate of 50 *kHz*.

### Stress-wave force balance technique setup

The SWT utilizes the structural vibration during short test times as the system’s dynamic response to determine the drag of the test model. The basic principle of the SWT is recovering the external loads from the measured strain signal through a deconvolution process with the system’s dynamic response function [8, 15]. The system’s dynamic response function can be obtained from dynamic calibration processes, which will be discussed in ‘**Dynamic calibration**’.

**Fig 5:**
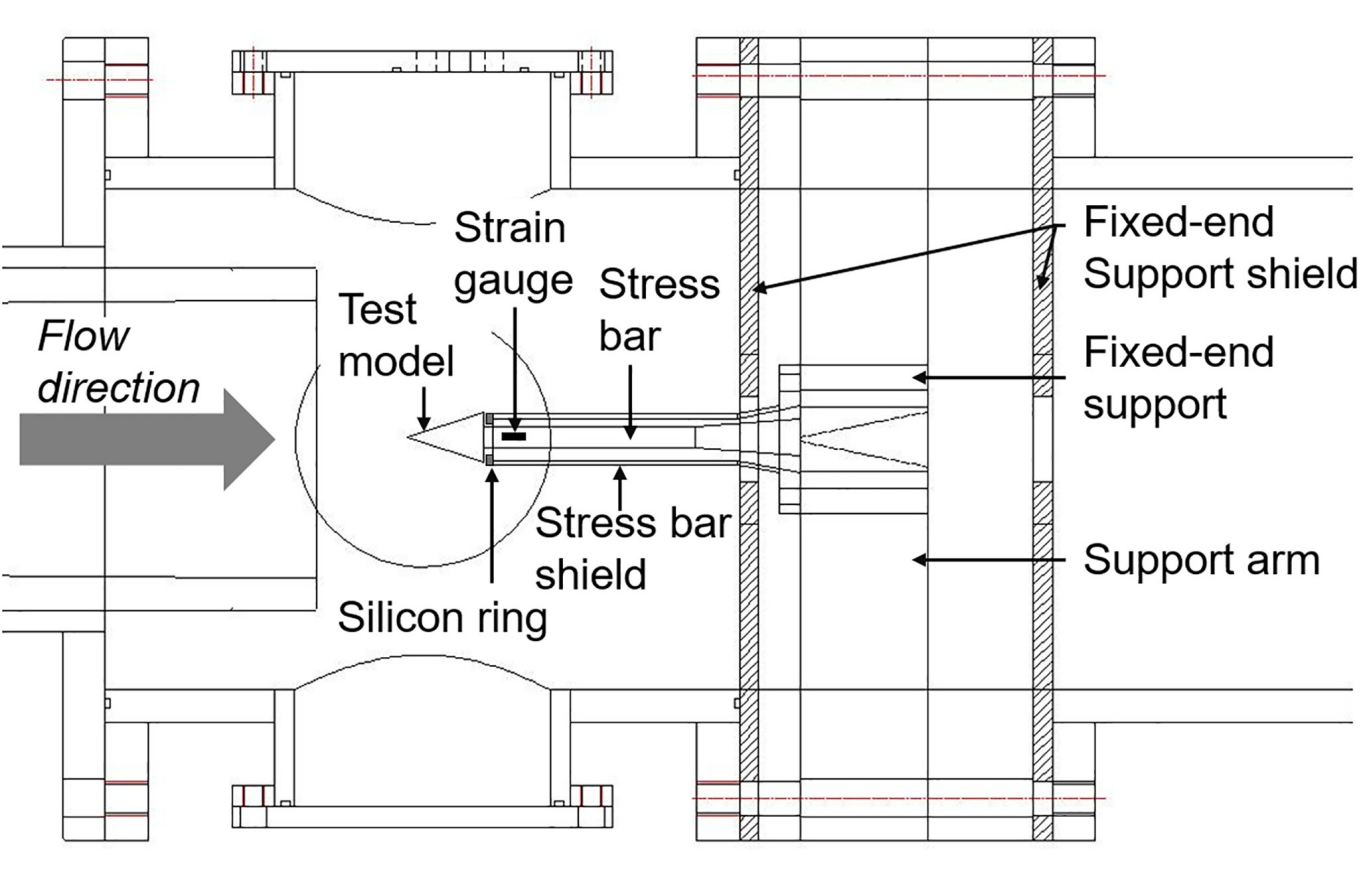
Schematic of the SWT setup.

Fig 5 shows a schematic of the SWT for drag measurements. The SWT consisted of a test model, a stress bar, and a fixed-end support. Each part of the entire system was manufactured from a single material, i.e., Al-6061. The length and the diameter of the stress bar were 150 and 10 *mm*, respectively. The test model and the stress bar were manufactured as a single integrated part to eliminate any vibration effects from bolts or welding. The stress bar was rigidly mounted to the fixed-end support and placed in the test section using four support arms. The fixed-end support arms had a wedged form in order to minimize test flow blockage and reflection of the shock wave. A semiconductor strain gauge (Model: S/UCP-120–090, Kulite), that can detect microlevel strains with reasonable accuracy [38], was attached parallel to the stress bar. The strain gauge and three 150 Ω resistors composed a quarter bridge outside of the test section. The resistance changes in the strain gauge caused by stress-wave propagation were transformed to voltage changes through the quarter bridge and recorded using an oscilloscope. The strain signal was recorded for 8 *ms* at a sampling rate of 1 *MHz*.

In order to separate the stress bar and the fixed-end support from the test flow, an aerodynamic shield was applied. The aerodynamic shield was sectioned into a stress bar shield and a fixed-end support shield, each made of Al-6061 and rubber, respectively. The outer diameter and the inner diameter of the stress bar shield were 22 and 20 *mm*, respectively, which were designed to be smaller than the diameter of the test model in order to prevent disturbance of the model base flow. A silicon ring was installed at the shield head to prevent a pressure build-up into the shield and contact problems of the shield to the model base. The gap between the test model and the silicon ring was 0.7 *mm*. The fixed-end support shield consisted of several rubber plates and was designed to wrap the support and four support arms. To avoid contact between the shield and the fixed-end support, the shield covered the system with a gap of 5 *cm*. The fixed-end support shield made with rubber plates was placed between the test section and the fixed end-support.

In this work, fixed-end support was thick (∼8 *cm*) and heavy (∼23 *kg*) in order to minimize signal disturbance. The disturbance effects, which could be caused by the vibration from the quarter bridge and the fixed-end configuration, and the methods to reduce these vibrations is discussed in ‘**Stress-wave force balance technique results**’.

### Numerical prediction

**Fig 6:**
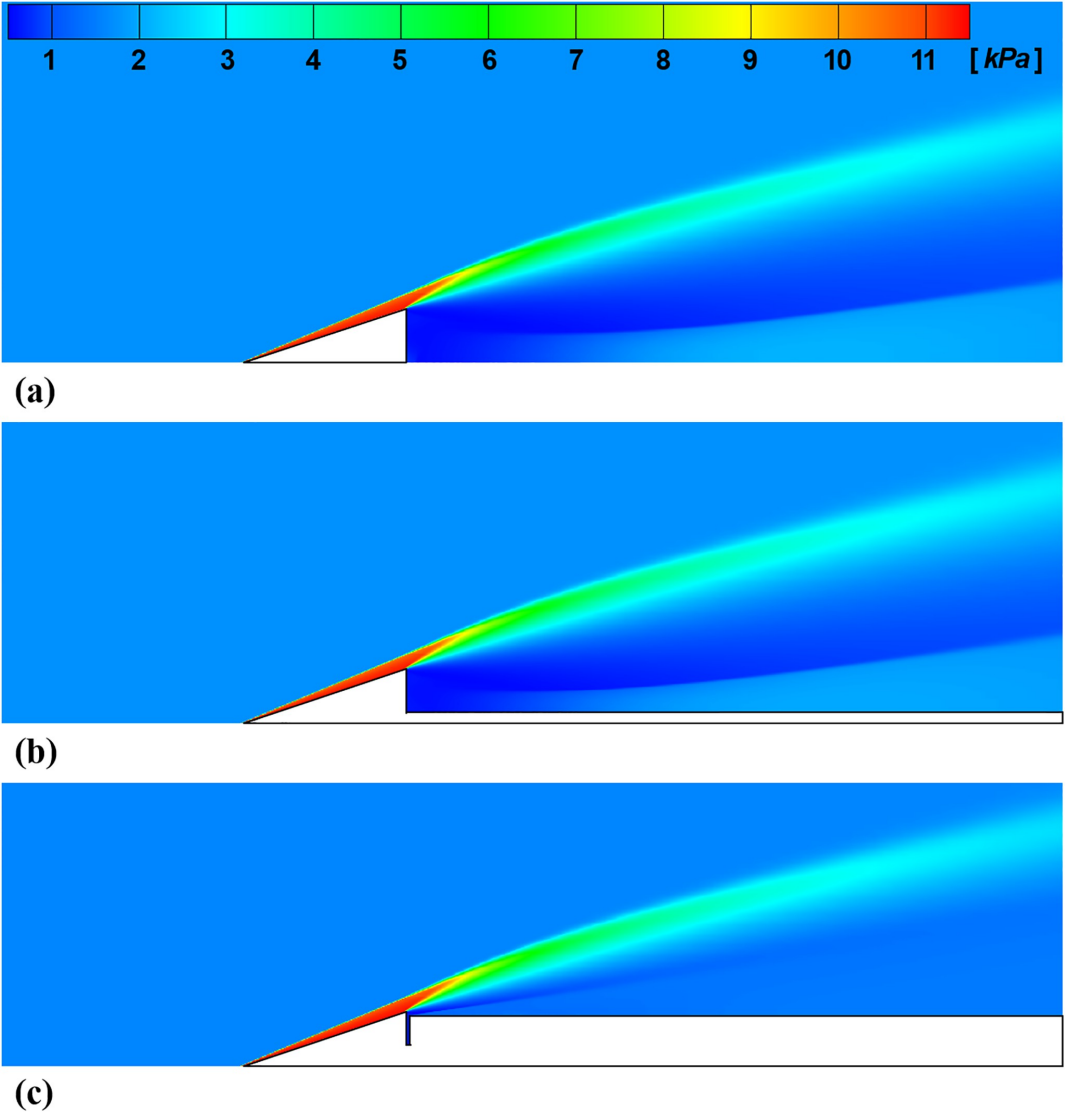
Calculated static pressure contours. (a) FFT, (b) MST, (c) SWT.

Fig 6 shows the pressure contours around the test model for three force measurement techniques predicted by the numerical analysis. In this work, the drag coefficient was predicted by numerical simulation using the commercial software ANSYS Fluent to compare the drag coefficient measurement results. The predicted results of the numerical simulation are referred to be the CFD results in the present work. Because of the axial symmetry of the model, the density-based axis-symmetric solver with the energy model was chosen for efficient calculation. In total, 112,700, 125,200, and 104,800 cell grids were used for the numerical simulation of the FFT, MST, and SWT, respectively. The MST’s cell grid was increased to calculate the boundary layer near the sting, and the SWT’s cell grid was decreased because of the large aerodynamic shied behind the test model. The convergence criterion of the calculation was a global residual value of 10^−6^. Since the Reynolds number around the test model, which can be calculated using the flow condition in Table 1 and the ideal gas relationship with the constant viscosity of the air, was 1.2×10^5^ with a length scale of the meter, the laminar model was used. Since the total temperature is below 2,000 *K*, a chemically non-reacting and ideal gas model was used. At the grid boundaries, a constant wall temperature of 300 *K* was used because of the short test time.

In Fig 6, an oblique shock wave with an angle of 22° is generated at the test model nose for all force measurement techniques. Behind the oblique shock wave, the flow was compressed, and the static pressure was increased approximately six times compared to the freestream. The flow pressure at the test model base was decreased under the freestream pressure because of the flow expansion at the vertex. Depending on the force measurement technique, the flow pressure behind the test model was different because of the different model-supporting mechanism. As the structure behind the test model base becomes large, the base pressure increases, and resulting in a decrease in drag. As a result, the total drag of the FFT, MST, and SWT, was obtained as 5.59, 5.55, and 5.37 *N*, respectively. However, the difference in drag due to the base pressure was relatively small within 5% because the pressure behind the shock wave in front of the test model, compared to the test model base pressure, is very high for the considered hypersonic flow condition. Because of the expansion fans near the test model base, the oblique shock wave was distorted, and the curvature of the shock wave was increased. The corresponding drag coefficient *C*_*D*_ of each technique was 0.283, 0.281, and 0.271 respectively. The details regarding the drag coefficient acquisition using the nozzle-exit flow condition is described in ‘**Drag coefficient acquisition**’.

## Data processing

### Dynamic calibration

For the MST and the SWT, which have the model supports to hold the test model, non-stabilized structural vibrations can be generated inside the model-support systems for a short test time. The non-stabilized structural vibrations can disrupt the values and the frequencies of the measured output signals, and consequently, can cause significant error in drag measurements. Therefore, for the drag measurements using the MST and the SWT, dynamic calibrations were performed to increase the accuracy of the drag measurements by compensating for the disturbance from the vibrations. The dynamic calibration is the process of obtaining each system’s ‘dynamic response function’ according to the external forces applied to the systems. The system’s dynamic response function is represented by the bandwidth of the vibrating frequencies and the shape of the system’s response. The basic principle of the drag measurement with dynamic calibration is to recover the drag acting on the system from vibrating signals using the system’s dynamic response function acquired through dynamic calibration.

If the system is assumed to be a linear time-invariant system, the following convolution integral is satisfied between the applied force input *I*, the system output *O*, and the system’s dynamic response function *g* [15, 36]:

$$\begin{array}{c} O\left(t\right)=\int_{0}^{t}g\left(t-\tau \right)I\left(\tau \right)\;d\tau , \end{array}$$ (1)

where *t* is the time variable and *τ* is the time shift dummy variable for the convolution integral. The system’s output signal *O* is the acceleration *a* for the MST or the strain *ε* for the SWT. If two of the three variables of *O*, *g*, and *I* are known, then the remaining variable can be determined using Eq 1. Therefore, for the dynamic calibration, the force input with a known shape, e.g., impulse or step, and amplitude is applied to the system, and the resulting system’s output signal can be measured. Because the force input and the resulting system’s output signal are known for the dynamic calibration, the system’s dynamic response function can be obtained from the deconvolution process between the force input and the system’s output based on Eq 1. Based on the fact that the system’s dynamic response function is independent to the size or shape of the force input applied to the system with the linear time-invariant assumption [8], the dynamic response function obtained through the dynamic calibration can be assumed to be the same as the system’s dynamic response function in the shock tunnel experiments. Consequently, the drag can be recovered by the deconvolution process based on Eq 1 between the obtained system’s dynamic response function and the system’s output signal measured from the shock tunnel experiments.

**Fig 7:**
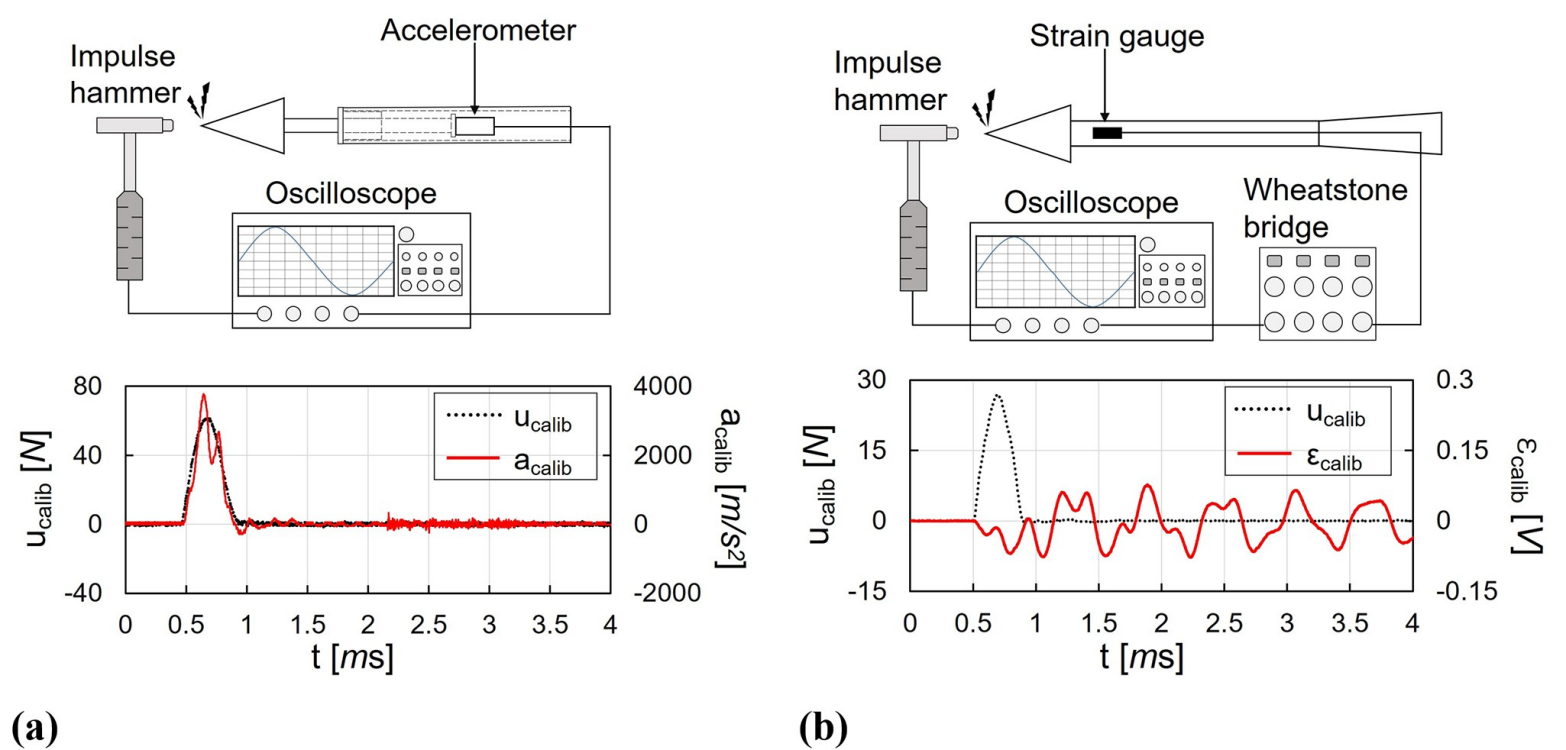
Dynamic calibration schematic and results. (a) MST, (b) SWT.

Fig 7 shows the schematics and the results of the dynamic calibration during 4 *ms* using the impulse hammer (Model: 086C01, PCB Piezotronics) for the MST and the SWT. Both results are represented by the applied force input *u*_*calib*_ with dotted black line and the system output with solid red lines (acceleration *a*_*calib*_ for the MST and strain *ε*_*calib*_ for the SWT). Subscript *calib* denotes the property obtained through the dynamic calibration. The acceleration signal of the MST is represented with *m*/*s*^2^, while the output of the SWT is denoted with *V*. Using the impulse hammer, the impulse loads were applied at the tip of the test model. The material of the hammer tip was plastic which has an adequate stiffness that can minimize the damage to the test model and provides impulses with a duration of approximately 0.5 *ms*. For the MST, the impulse input time histories, which were obtained using the internal piezoelectric element, and resulting acceleration signals were simultaneously recorded on the oscilloscope, as shown in Fig 7(a). For the SWT, the strain signal processed by the quarter bridge was recorded as the system’s output (Fig 7(b)). Consequently, the systems’ dynamic response function could be obtained using least-squares deconvolution [39, 40] between the impulse hammer inputs and the output of the systems. Since the electrical noise can cause errors in the deconvolution process [41], a fifth-order Savitzky-Golay filter [42] with a length of 31-data points was applied to all recorded data to eliminate noise with high frequencies.

When comparing the two dynamic calibration results in Fig 7, it can be observed that the MST and the SWT had different characteristics in the system’s outputs. For the MST in Fig 7(a), the changes in acceleration *a*_*calib*_ was similar to the impulse input *u*_*calib*_; the acceleration increased and decreased with the impulse input rise and drop, and stabilized around the zero level after the impulse duration. At 0.75 *ms* in Fig 7(a), the acceleration fluctuates by the stress wave propagation in the model-sting. The vibration with small amplitude found after the impulse duration was induced from the friction effect between the linear ball bush and the sting. Because the stress wave propagation and reflection were limited only in the model-sting system for the MST, the stress wave effects were quickly stabilized and only observed during the impulse duration. However, for the SWT in Fig 7(b), the trace and the shape of the strain gauge signal, i.e. strain *ε*_*calib*_, and the impulse input *u*_*calib*_ were significantly different. In addition, the output for the SWT vibrated with low frequencies, even after the impulse duration, because the stress wave propagation was not stabilized during the short time and induces the response of the strain after the impulse duration. Therefore, the system’s output should be measured for a more extended period than the impulse duration in the SWT since the accuracy of the SWT is strongly depend on the accuracy of the dynamic calibration and the obtained system’s dynamic response function.

### Drag coefficient acquisition

In order to compare the drag measurement results from three drag measurement techniques, the drag coefficient was calculated from the measured drag using the flow condition at the nozzle exit in Table 1. The drag coefficient for the supersonic flow field *C*_*D*_ can be calculated as follows using ideal gas equation and Rayleigh pitot relation [33]:

$$\begin{array}{c} C_{D}\equiv \frac{D}{\frac{\gamma }{2}P_{\infty }M_{\infty }^{2}A}=\frac{D{\left[\left(\gamma +1\right)\frac{M_{\infty }^{2}}{2}\right]}^{\frac{\gamma }{\left(\gamma -1\right)}}}{\frac{\gamma }{2}P_{pt}{\left[(\frac{2\gamma M_{\infty }^{2}}{\gamma +1})-(\frac{\gamma -1}{\gamma +1})\right]}^{\frac{1}{\gamma -1}}M_{\infty }^{2}A}, \end{array}$$ (2)

where *D* is the drag, *γ* is the specific heat ratio, *A* is the cross-sectional area. It can be seen in Eq 2 that the drag coefficient is a function of the experimentally measurable quantities of drag *D*, nozzle-exit pitot pressure *P*_*pt*_, and freestream Mach number *M*_∞_.

**Fig 8:**
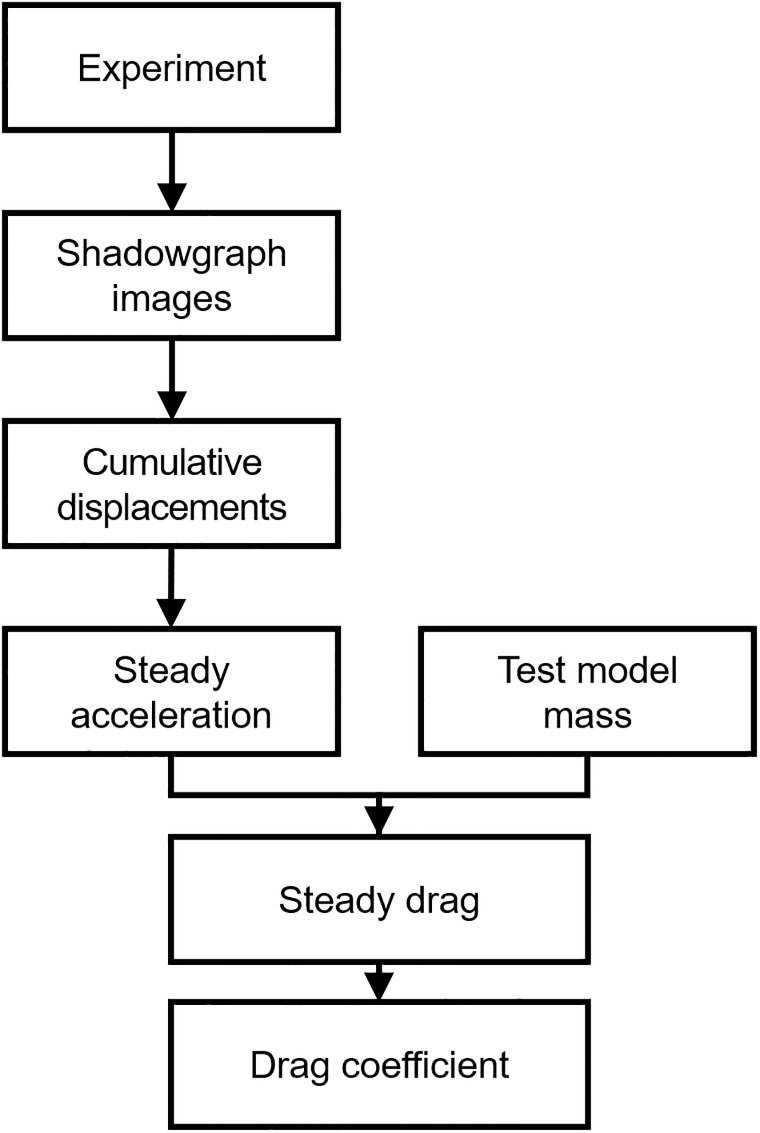
Drag coefficient acquisition using the FFT.

**Fig 9:**
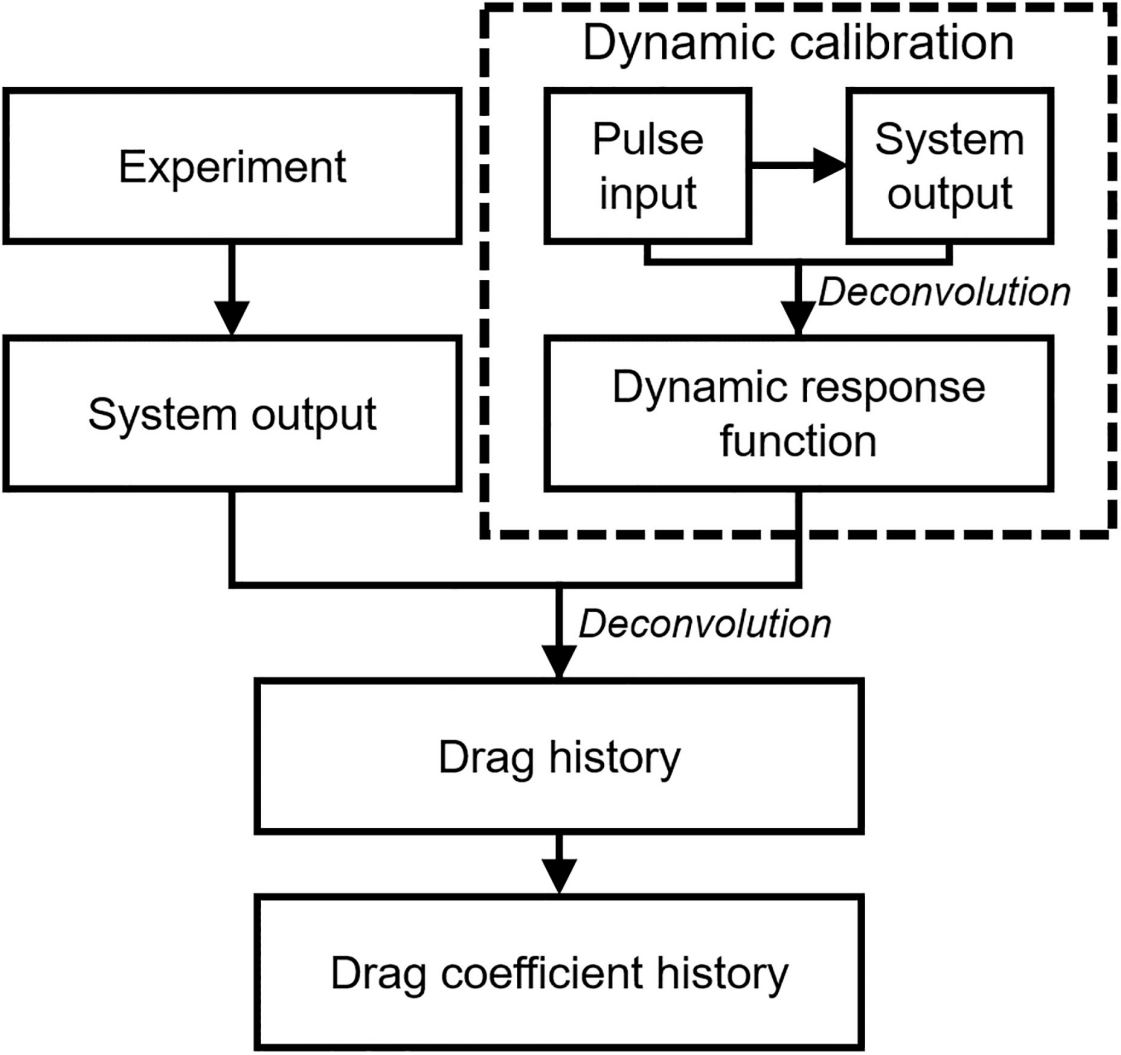
Drag coefficient acquisition using the MST and the SWT.

Figs 8 and 9 show the drag coefficient acquisition procedure for the FFT, and for the MST and the SWT, respectively. Since the FFT, MST, and SWT have different model-supporting mechanisms and measuring processes of the physical quantities to determine the drag coefficients, the drag coefficient acquisition process differed depending on the technique. In the FFT, where the drag was measured from displacement of the free-flying test model with detachable wires, calibration of the non-stabilized structural vibrations inside the model-support system was not required. Unlike the FFT, the MST and SWT had supports that weakly or rigidly hold the test model, so that the drag coefficients should be determined by dynamical calibration of the non-stabilized structural vibrations in the measured signal.

For the drag coefficient acquisition procedure in the FFT of Fig 8, the test model’s cumulative displacements during steady time were extracted from the shadowgraph image using the corner detector. Then, the acceleration of the test model in a steady-state was determined from the polynomial curve-fit model of the test model’s cumulative displacements. With the mass of the test model, the drag in a steady-state was calculated from the acceleration using Newton’s second law of motion. Finally, the drag coefficients could be obtained from the drag using Eq 2.

Fig 9 shows the drag coefficient acquisition procedure for using the MST and the SWT where the dynamic calibration was considered. The dynamic calibration and the shock tunnel experiment were separate procedures. In the present work, the dynamic calibrations of the MST and the SWT were performed before the shock tunnel experiments, and the dynamic response function of each technique was obtained. Based on the systems’ dynamic response function obtained from the dynamic calibrations, the drag histories of the shock tunnel experiments were recovered using the deconvolution process. Because the drag of the MST and the SWT were recovered through the time deconvolution process, the recovered drag histories throughout time could be obtained in the MST and SWT, unlike the steady drag values for the FFT. After the drag recoveries, similar to the FFT tests, the histories of the drag coefficients could be obtained using the flow condition at the nozzle exit and Eq 2.

## Results and discussion

### Free-flight technique results

**Fig 10:**
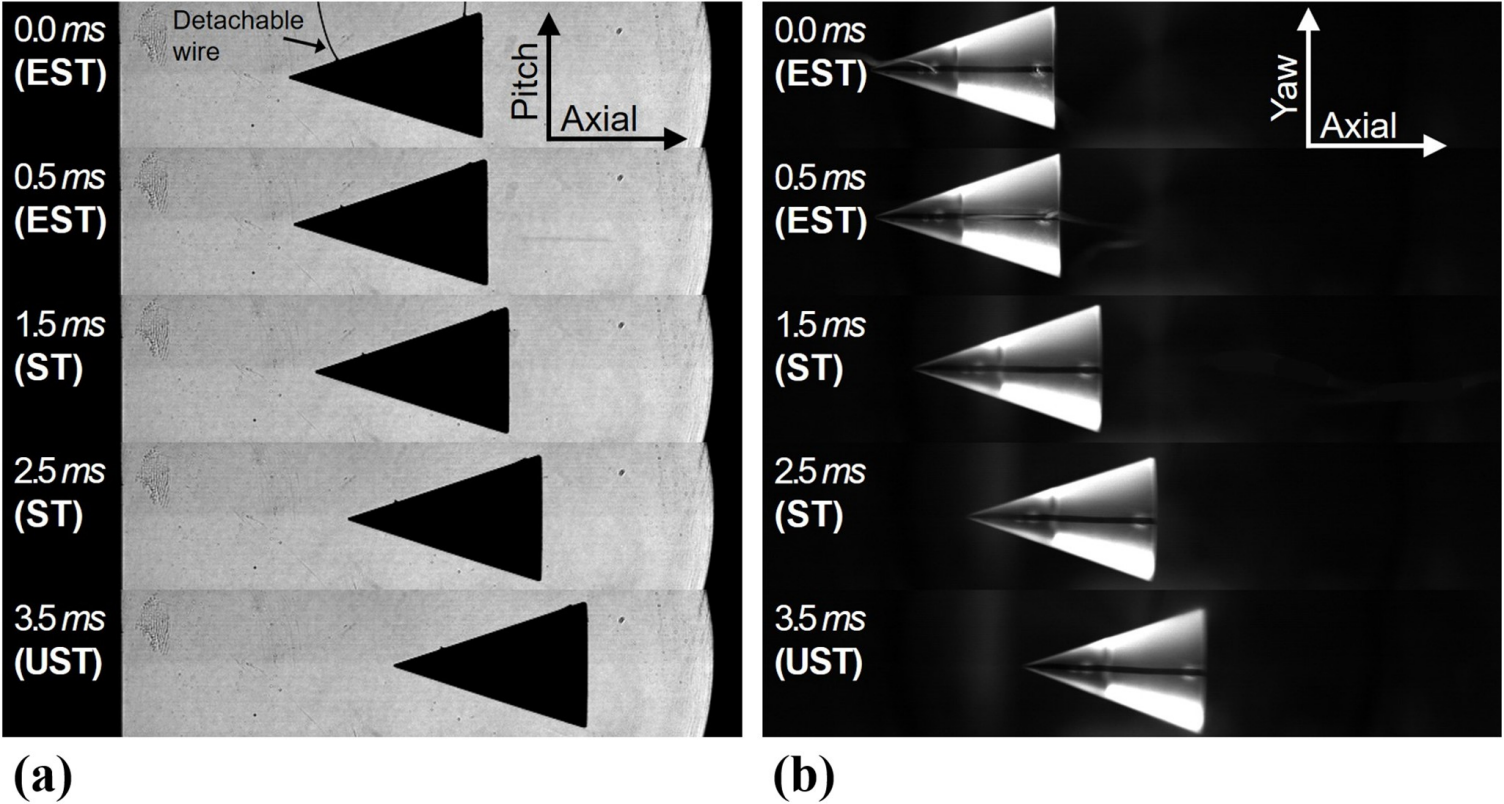
Captured images of the free-flying test model in the FFT. (a) Axial and Pitch directions, (b) Axial and yaw directions.

Fig 10 shows representative images of the free-flying model’s trajectory using the FFT. The abbreviated terms EST, ST, and UST in the images denote the flow establishment, steady flow, and unsteady flow, respectively. The start timing of the FFT (0.0 *ms*) was synchronized to the pitot pressure history at the nozzle exit (0 *ms* in Fig 2) in order to identify the test flow development in the free-flying model’s images. When the shock wave reached the test model, the wires were detached from the model surface, and the test model freely flew in the flow direction. As mentioned, the images that confirmed only the pure axial motion were used to determine the drag for the FFT.

Fig 10(a) shows the shadowgraph images in the axial and pitch directions captured by the side camera in Fig 3(b). Because the shadowgraph technique visualizes the density change in the field of view, the test model is presented with the black triangle. In Fig 10(a), the wires were detached from the test model after the test flow arrival at the test model and escaped the field of view. Fig 10(b) shows the directly captured model trajectories in the axial and yaw directions with the upper camera in Fig 3(a). For the directly captured images, the test model made was presented transparently, and it was difficult to identify the detachable wires because of the low contrast of the images. A line at the test model surface was the guideline for the wire attachment location. It can be observed that the test model flies along the axial direction, and the displacements along the pitch and the yaw directions were negligible.

**Fig 11:**
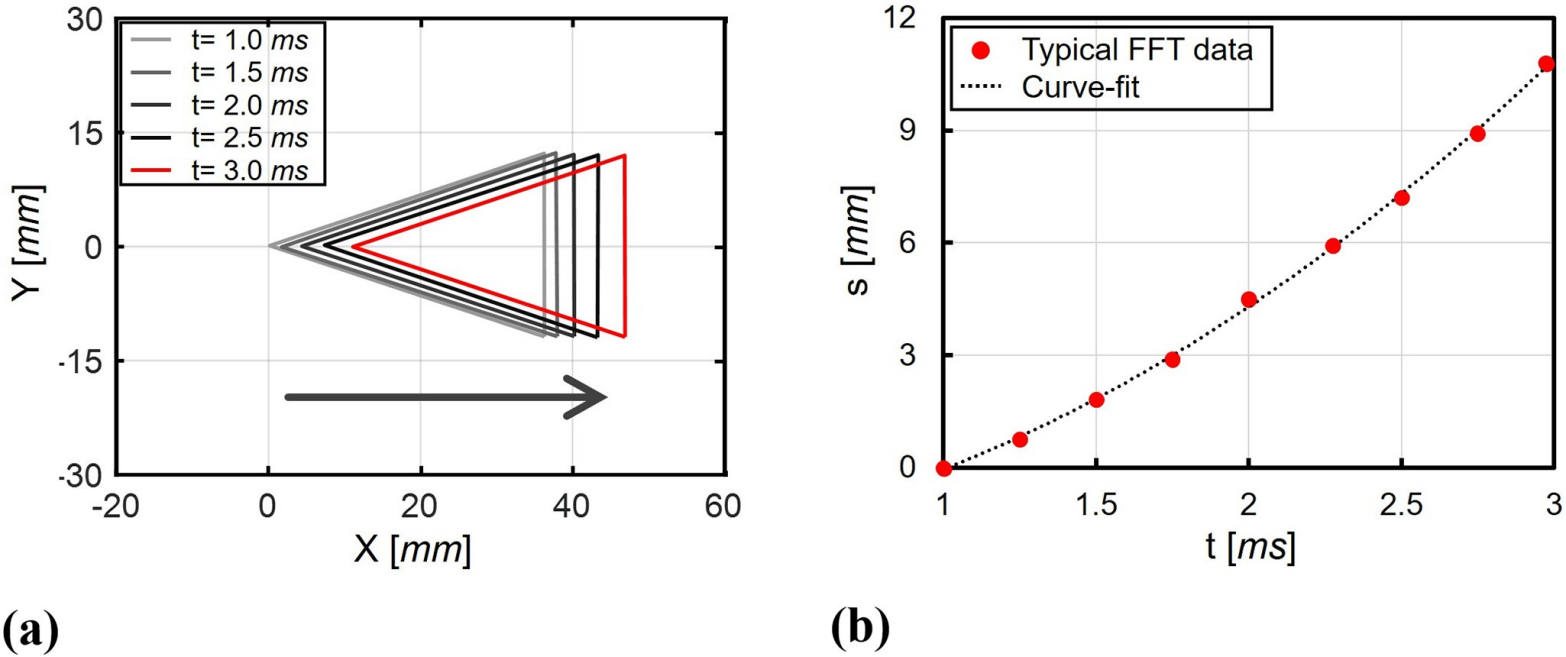
Corner detection results of the free-flying model in the FFT. (a) Typical detected corners, (b) Cumulative displacements.

Fig 11 represents the corner detection results of the shadowgraph images in Fig 10(a) and the cumulative displacements *s* of the test model along the axial direction. In Fig 11(a), the five representative detection results with an interval of 0.5 *ms* are shown. The horizontal axis *X* represents the axial direction and the vertical axis *Y* represents the pitching direction. It can be seen that the test model was moved along the axial directions over time because of the drag. A total of 80 images were used for the corner detection to obtain the cumulative displacements and the change in the angle-of-attack. In order to minimize the disturbance effects caused by the initial flow development and the unsteady flow termination, the middle of the steady test time (from 1 to 3 *ms*) were used.

In this work, the axial displacements of the test model was represented by the axial displacements of the test model’s center-of-mass. The location of the test model’s center-of-mass could be obtained from the two-thirds point at the test model’s axis. After obtaining the test model’s cumulative displacements, the polynomial curve-fit model was applied to the cumulative displacements, and the acceleration of the test model was determined from the second derivation of the curve-fit model. For the present FFT, quadratic polynomial curve-fit model was used assuming a constant acceleration of the test model during the short-duration test time. In order to verify the pure axial motion of the test model, the change of the test model’s angle-of-attack was determined. The test model’s angle-of-attack could be obtained from the angle between the horizontal line and the line passing through the test model’s center-of-mass and nose. The average test model’s angle-of-attack during the analysis time was measured as -0.345° ± 0.1° for Fig 11, which indicates that the test model’s angle-of-attack could be assumed to be zero. It was confirmed from the angle-of-attack results that the test model has pure axial motion during the analysis time.

Fig 11(b) shows the cumulative displacements *s* and its polynomial curve-fit model using the quadratic regressions. The axial displacements of the free-flying model during analysis time was approximately 11 *mm*, and the FFT data was well-matched with the quadratic curve-fit model. From the quadratic curve-fit model of the cumulative displacement in Fig 11(b), the average acceleration of the test model was determined as 2,007 *m*/*s*^2^ and accordingly, the average drag was 5.44 *N* in the FFT. Consequently, the average drag coefficient of the FFT was obtained as 0.284. The relative difference between the drag coefficients of the FFT and the CFD results was 0.35%.

### Movable-support force balance technique results

**Fig 12:**
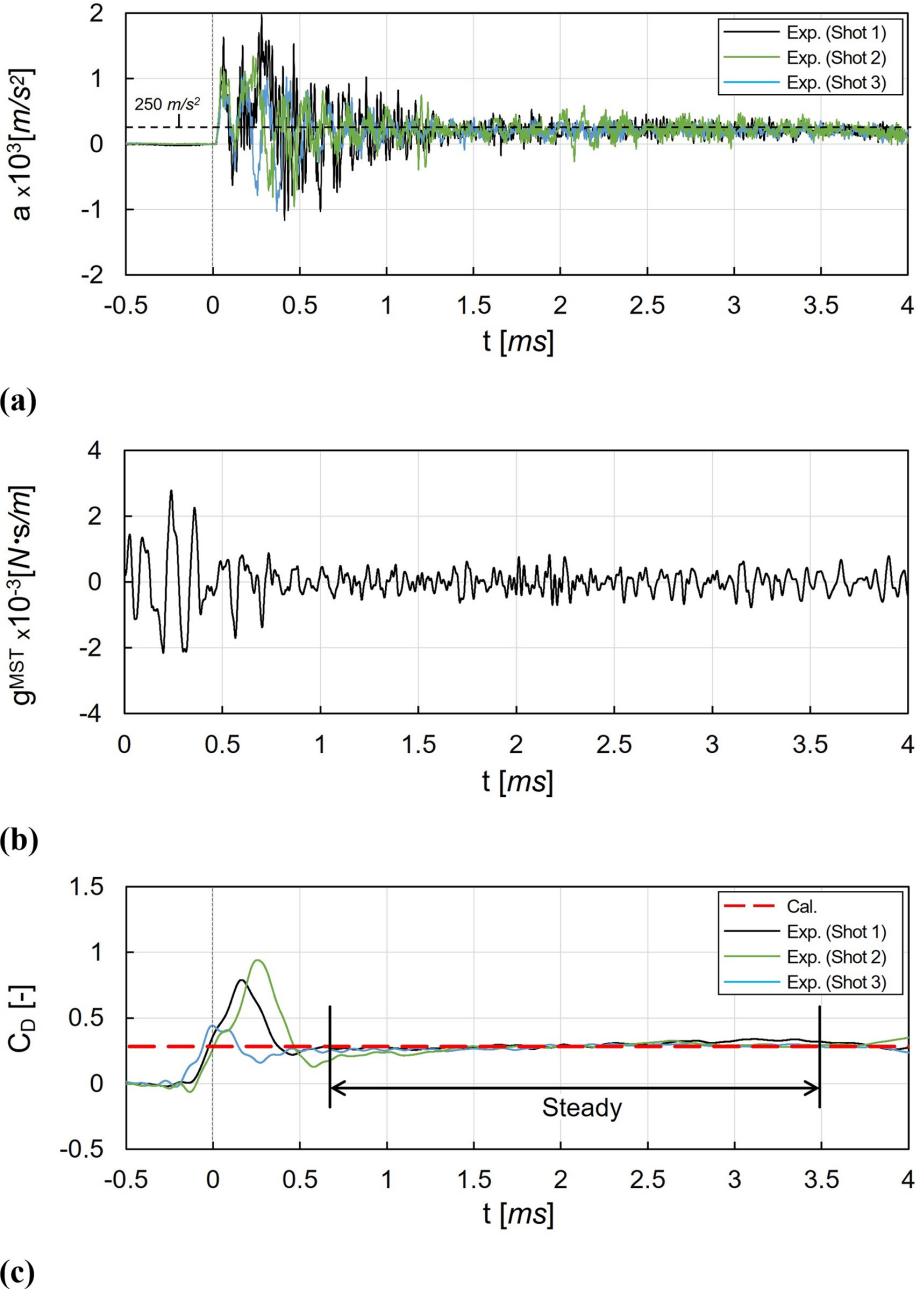
Drag coefficient measurements using the MST. (a) Measured acceleration, (b) Dynamic response function of the MST for 4.0 *ms*, (c) Recovered drag coefficient histories.

Fig 12 shows the results of the drag coefficient measurement using the MST. In Fig 12(a), the direct measurement results for acceleration *a* using accelerometer in shock tunnel experiments is presented. Three multiple shots showed similar acceleration signals. The measured accelerations rose after the test flow arrival at 0 *ms* and vibrated significantly until 0.5 *ms*. The vibration in the accelerations was reduced, and the steady-state of the acceleration was maintained until the decrease after 3.5 *ms*. The initial vibrations until 1.0 *ms* were possibly originated from the stress wave propagation inside the model-sting system, which could be generated by the sudden aerodynamic application occurred from the sudden pressure rise of the nozzle-exit pitot pressure *P*_*pt*_ at 0.25 *ms* in Fig 2. After 2 *ms*, the acceleration was stabilized and vibrated at approximately 250 *m*/*s*^2^ with a relatively weak amplitude. The vibrations with relatively small amplitudes observed from 2 to 3.5 *ms* were originated from the movement of the sting through the linear ball bush.

Fig 12(b) shows the MST’s dynamic response function *g*^*MST*^, which could be obtained from the dynamic calibration in Fig 7(a). Similar to Fig 12(a), a significant vibrations were observed in the dynamic response function from 0 to 0.5 *ms*. After 1.0 *ms*, the dynamic response function was stabilized.

Fig 12(c) shows the drag coefficient histories, which was obtained from deconvolution process using acceleration histories in Fig 12(a) and dynamic response function for the MST *g*^*MST*^ in Fig 12(b) as inputs. The drag coefficient *C*_*D*_ rose before 0 *ms* because of the signal smoothing in deconvolution. It can be seen that the vibrations until 1 *ms* were reduced, comparing to the acceleration histories in Fig 12(a). The time required for the stabilization in the drag coefficient *C*_*D*_ was longer than that in the nozzle-exit pitot pressure *P*_*pt*_ in Fig 2. This can be explained by the stabilization of the recirculation zone between the test model and the aerodynamic shield. Because the establishment of the pressure drag acting on the test model could be delayed by the sudden pressure increase at the test model base during the stabilization of the recirculation zone.

For the drag coefficient acquisition in the MST, the steady time was set as 0.65 to 3.5 *ms*, and the steady-state drag coefficient was 0.301. The relative difference between the drag coefficients of the MST and the CFD results was 6.64%.

### Stress-wave force balance technique results

**Fig 13:**
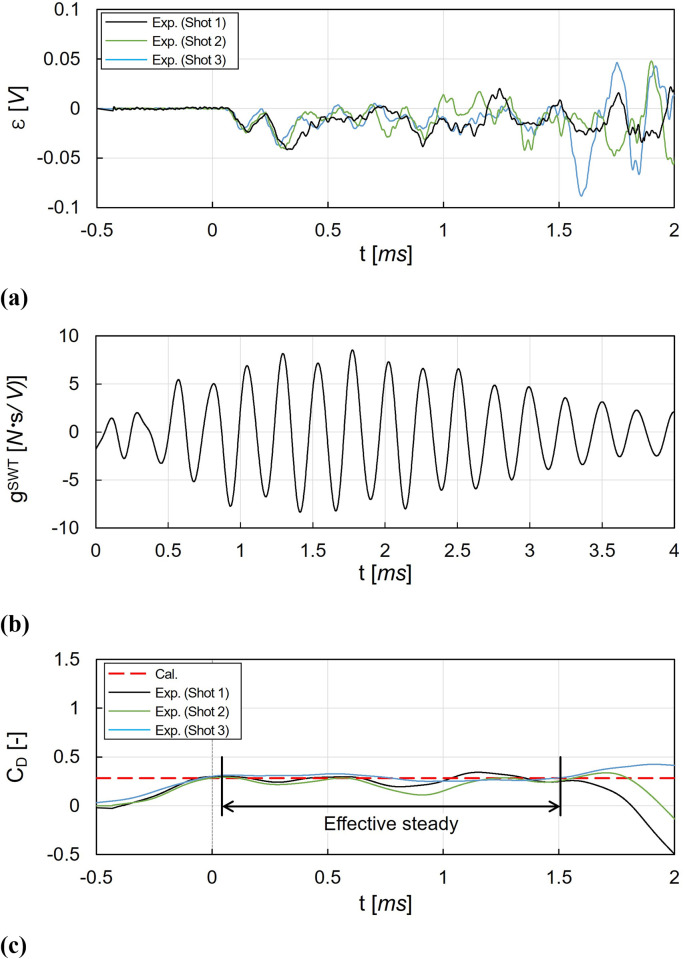
Drag coefficient measurements using the SWT. (a) Measured strain, (b) Dynamic response function of the SWT for 4.0 *ms*., (c) Recovered drag coefficient histories.

Fig 13 shows the results of the drag coefficient measurement using the SWT. Fig 13(a) shows the measured strain *ε* histories until 2 *ms*. Compared to the measured acceleration histories of the MST in Fig 12(a), the following features can be observed in the strain histories. First, the response of strain was slower than that of acceleration. The initial changes in the strain started at approximately 0.05 *ms*, which was far later than the start of the acceleration histories with 0 *ms*. In addition, there were no sudden rises in the initial strain. This is because it took time for the stress wave to propagate and reach the strain gauge. Second, the vibration of the strain had a relatively small amplitude. Because, in the present work, the rigid support made with aluminum alloy was used and the drag acting on the test model was relatively small (approximately 6 *N*), the system’s deformation originated from the drag was smaller than the acceleration for the MST. Finally, the strain was significantly disturbed with vibrations with a significant amplitude after 1.5 *ms*. The strain disruption was mainly originated from the unexpected vibrations, which will be discussed latter in this section. Because of the strain disturbance the effective steady time of the SWT was set as 0.05 to 1.5 *ms*.

Fig 13(b) shows the dynamic response function for the SWT *g*^*SWT*^ which was obtained from deconvolution in the dynamic calibration. The dynamic response functions for the SWT and that for the MST were significantly different. In the SWT, the dynamic response function had continuous vibrations within the entire measurement time with a low frequency.

Fig 13(c) shows the drag coefficient histories, which was obtained from deconvolution using the strain histories in Fig 13(a) and the dynamic response function for the SWT in Fig 13(b). As shown in the strain, the recovered drag coefficients were also disrupted after 1.5 *ms*, and only the drag coefficients before 1.5 *ms* were used for the determination of the average value. Unlike the MST, the overall histories of the drag coefficients were smooth because the SWT was used the strain with a slower response to determine the drag. The rise in the drag coefficients began before 0 *ms*, resulting from signal smoothing through the deconvolution process. During the effective steady time (from 0.05 to 1.5 *ms*), the average drag coefficient for the SWT was 0.265, and the relative difference between the drag coefficients of the SWT and the CFD results was -2.26%.

For the SWT, the effective steady time was terminated in Fig 13(a) and 13(c) by the vibrations with significant amplitudes. The signal disruptions by the vibrations were possibly originated from 1) the propagation of the test facility-driven vibrations, and 2) the bending strain domination. Because the minimization of the signal disruptions is recommended for a higher accuracy of the SWT, the methods for reducing the disruptions are suggested as follows. First, in order to minimize the propagation of the external vibrations from the test facility or the test section to the system, a vibration isolation system design can be considered. The vibration isolation can be achieved by the free-end configurations using thin wires or threads to isolate the system from the horizontal planes’ external vibrations [8, 9, 14, 15, 22, 43]. Springs or rubber pads can be also applied for the fixed-end configuration to reduce the propagation of the external vibrations [44, 45]. Second, a bending strain compensated system can be used for the reduce the bending strain effects, which is much larger than the axial strains for typical load distribution, and induces significant errors in the drag measurements. For bending compensation of the stress bar, two strain gauges mounted with the diametrically opposite positions at the stress bar can be used to compose a half Wheatstone bridge [14, 15]. The hollow tubing stress bar design can also be considered to increase the bending stiffness compared to the axial stiffness in order to minimizing the bending effect [8].

### Uncertainty analysis

The drag coefficients were acquired from the various measured properties, as shown in Eq 2. Each measured property had uncertainties that were combined and propagated to the uncertainty of the drag coefficient acquisition. Therefore, in order to estimate the total uncertainty of the drag coefficient and the proportion of each property’s uncertainty on the total uncertainty of the drag coefficient, an uncertainty analysis was performed.

The total uncertainty of the drag coefficient can be calculated from the measurement uncertainties of the fundamental properties, using the error propagation method [46]. In this work, the nozzle-exit Mach number *M*, nozzle-exit pitot pressure *P*_*pt*_, and measured drag *D* were considered as the fundamental quantities. Assuming each fundamental quantity is independent to each other and normally distributed, the total relative uncertainty of the drag coefficient $X_{C_{D}}$ can be calculated as follows:

$$\begin{array}{c} X_{C_{D}}^{2}={\left(\frac{\left(\frac{\partial C_{D}}{\partial M_{\infty }}\right)}{C_{D}}\delta M_{\infty }\right)}^{2}+{\left(\frac{\left(\frac{\partial C_{D}}{\partial P_{pt}}\right)}{C_{D}}\delta P_{pt}\right)}^{2}+{\left(\frac{\left(\frac{\partial C_{D}}{\partial D}\right)}{C_{D}}\delta D\right)}^{2}, \end{array}$$ (3)

where *δM*_∞_ is the uncertainty of the Mach number measurements, *δP*_*pt*_ is the uncertainty of the nozzle-exit pitot pressure measurements, and *δD* is the uncertainty of the drag measurements. For the uncertainties of measurements of Mach number, nozzle-exit pitot pressure, and drag, the standard errors based on multiple measurements were used. The partial derivatives of the drag coefficients regarding each property, $\frac{\partial C_{D}}{\partial M_{\infty }}$, $\frac{\partial C_{D}}{\partial P_{pt}}$, and $\frac{\partial C_{D}}{\partial D}$, were derived from Eq 2. Because the absolute uncertainties of each property had different units and values and were difficult to compare directly, the total uncertainty of the drag coefficient was divided by the drag coefficient to have relative value.

Table 2 represents the results of the uncertainty analysis of the drag coefficients. Each uncertainty terms in Eq 3 of the Mach number $\left(\frac{\partial C_{D}}{\partial M_{\infty }}/C_{D}\right)$, the nozzle-exit pitot pressure $\left(\frac{\partial C_{D}}{\partial P_{pt}}/C_{D}\right)$, and the drag $\left(\frac{\partial C_{D}}{\partial D}/C_{D}\right)$ are represented separately, in order to identify the proportions of each uncertainty on the total uncertainty of the drag coefficient. The uncertainties of the Mach number measurements and the nozzle-exit pitot pressure measurements had constant values, regardless of the drag measurement techniques. This is because the test flow diagnosis, which includes the Mach number and the nozzle-exit pitot pressure measurements, were performed before the drag measurements without the drag measurement technique setup and were independent of the drag measurement technique.

**Table 2:** Uncertainty analysis of the drag coefficient measurements.

Tech.	*C*_*D*_ [-]	$\left|(\frac{\partial C_{D}}{\partial M_{\infty }}/C_{D})\delta M_{\infty }\right|$	$\left|(\frac{\partial C_{D}}{\partial P_{pt}}/C_{D})\delta P_{pt}\right|$	$\left|(\frac{\partial C_{D}}{\partial D}/C_{D})\delta D\right|$	$|X_{C_{D}}|$
FFT	0.284	6.37%	0.88%	5.67%	8.57%
MST	0.301	6.37%	0.88%	4.20%	7.68%
SWT	0.265	6.37%	0.88%	4.47%	7.83%

Comparing the proportion of Table 2, the proportion of each property’s uncertainty on the drag coefficients’ uncertainty is reduced in the order of the Mach number $\left|(\frac{\partial C_{D}}{\partial M_{\infty }}/C_{D})\delta M_{\infty }\right|$, the drag $\left|(\frac{\partial C_{D}}{\partial D}/C_{D})\delta D\right|$, and the nozzle-exit pitot pressure $\left|(\frac{\partial C_{D}}{\partial P_{pt}}/C_{D})\delta P_{pt}\right|$. The uncertainties for each property originated from the measurement process.

For the Mach number measurement, the uncertainty was mainly caused by the imaging process. Because the Mach number dominates the structure of the supersonic flow field and it is difficult to measure the pre and post-shock wave flow conditions simultaneously, it is general to determine the Mach number by using the flow visualization techniques to capture the angle of the oblique shock wave [34] or the shock wave standoff distance at the blunt body [31]. In the present work, the flow visualization technique, the Z-type shadowgraph technique, was also used to determine the Mach number. In the shadowgraph images with a resolution of 1,280×120 *pixels*, the diameter of the 3 *cm* hemisphere was counted as 128 *pixels*, and the shock wave standoff distance in front of the hemisphere was approximately 14 *pixels*, with a resulting shock wave standoff distance-hemisphere diameter ratio of approximately 0.109. Because of the very small value of 0.109, the shock wave standoff distance-hemisphere diameter ratio could be varied by more than 7% with one pixel-level uncertainty of the ratio. Therefore, the uncertainty of the Mach number measurements was the largest. On the other hand, the uncertainty of the nozzle-exit pitot pressure measurements was the lowest because the pitot pressure at the nozzle exit was measured directly, without any corrections or conversions.

The proportion of the drag measurements’ uncertainty on the drag coefficients’ uncertainty $\left|(\frac{\partial C_{D}}{\partial D}/C_{D})\delta D\right|$ was decreased in the order of the FFT, SWT, and MST. Because the uncertainties of the Mach number and the nozzle-exit pitot pressure measurements were independent of the drag measurement technique, the total relative uncertainty of the drag coefficient $|X_{C_{D}}|$ was also decreased in order of the FFT, SWT, and MST.

Because of the difference in the drag acquisition process of each technique, the main cause of the uncertainties of the drag measurements was different for each technique. For the FFT, the main source of the measurement uncertainty was the corner detection uncertainty of the optical tracking method. The corner detection with the optical tracking method is generally used in the FFT for obtaining the displacements of the free-flying model because it has difficulty installing accelerometers inside the small and light model. In determining the acceleration of the test model from the polynomial curve-fit model of the detected displacements, one pixel-level uncertainty in the captured displacements could distort the entire curve-fit model, and a significant measurement uncertainty could be induced. Consequently, the FFT had the largest uncertainty among the three techniques.

The uncertainty of the SWT, which was possibly induced by unexpected and uncompensated vibrations, was the second largest. Because the SWT is an inverse technique based on the system’s dynamic response function obtained from the dynamic calibration, the SWT uncertainty can be amplified if the strain of the shock tunnel experiments includes some vibrations which are not considered in the dynamic calibration. In the present SWT, the bending strain and external vibration could cause significant disturbances in the drag coefficient acquisition process. Compared to the FFT without calibrations, some portions of the disturbance were calibrated, and the resulting measurement uncertainty of the SWT was smaller than that of the FFT.

The MST had the lowest uncertainty. It can be seen that the movable support of the present MST had the free-end configuration because the support did not constrain the motion of the model with the sting along the axial direction. For the supports with the free-end configuration, the propagation of external vibrations through the support to the system could be minimized. Furthermore, in the present MST, the internal vibration of the system, such as friction between the ball bush and sting, was calibrated through the dynamic calibration; therefore, the MST has the lowest uncertainty.

In order to reduce the total relative uncertainties for the drag coefficient measurement, the following methods can be considered. For the reduction of the Mach number measurement’s uncertainty, which was the largest and independent of the drag measurement techniques, a shadowgraph imaging setup with a higher resolution can be used to reduce the pixel-level uncertainty. For higher resolution images, the absolute amount of the pixel for the same length of the test model is increased and the uncertainty of the pixel-level uncertainty can be decreased relatively. The high resolution of the images can be achieved by reducing the capturing speed of the flow visualization setup or increasing the diameter of the hemisphere model or the post-processing method based on the deconvolution [47].

For the reduction in the FFT uncertainty, which is the largest among the drag measurement techniques, the increase of the detected images’ resolution and the test model acceleration can be considered. As mentioned in the uncertainty of the Mach number measurement, the increase of the detected images’ resolution can be achieved by reducing the capturing speed or using the post-processing method of images. The test model’s acceleration of the FFT can be increased by using a larger test model made with low-density material which increasing the cross-sectional area and reducing the mass of the test model. The SWT uncertainty can be reduced by the compensation or the reducing of the bending strain and external vibration. As described in ‘**Stress-wave force balance technique results**’, bending strain can be compensated using the half Wheatstone bridge. For the reducing of external vibrations, vibration isolation system using the free-end configuration can be considered.

### Comparison of the test environment between drag measurement techniques

In this section, the drag measurement system setup of the present work and the available literature are compared to estimate the appropriate test environment range of the application of drag measurement techniques of the FFT, MST, and SWT. The comparison can be used as a referable data in selecting the appropriate drag measurement techniques for the specific test environments.

In the present work, the test environments for the drag measurement techniques were represented with the size of the test model, mass and length, and the flow establishment time around the test model. For the size of the test model, the maximum mass of the test model is directly determined by model-supporting mechanism, which differs in the drag measurement techniques. In the present work, the size factor *ξ* was defined as the mass compensated by the axial length as follows:

$$\begin{array}{c} \xi \equiv (\frac{m}{l}), \end{array}$$ (4)

where *m* is the mass, and *l* is the axial length of the test model.

The flow establishment time around the test model is related to how fast the response time of the measurements is. If the flow establishment time is much longer than the response time of the drag measurement technique, the unexpected response of the test model-support system can be induced by the unstable aerodynamic force application during the flow development time, which significantly drops the accuracy of the drag measurement. In the present work, the flow establishment time around the test model *t*_*e*_ for the attached flows was approximated as follows [5]:

$$\begin{array}{c} t_{e}\approx 10\frac{l}{v_{\infty }}, \end{array}$$ (5)

where *v*_∞_ is the freestream velocity. By using the size factor *ξ* and the flow establishment time *t*_*e*_, the differences in the setup between the drag measurement technique can be compensated, and direct comparisons can be achieved.

Table 3 shows the present work’s test model size, freestream velocity, and the resulting size factor and flow establishment time of the FFT, MST, and SWT. The mass of the FFT, MST, and SWT was different for the present work. This is because the test model in the FFT was made of low-density material (acrylic), while both the MST and the SWT were made with high-density material (aluminum). Comparing the MST and the SWT, the SWT had a relatively heavier mass because of the larger diameter of the sting behind the test model. The axial lengths of the test model are the same because the test model geometry for the three techniques was identical. Because of the mass, the size factor also increased in the order of the FFT, MST, and SWT. For the flow establishment time, three drag measurement techniques have the same flow establishment time since the drag measurements were performed in identical test flow condition.

**Table 3:** Logarithmic size factor and flow establishment time of the present work.

Tech.	*m* [*g*]	*l* [*mm*]	*v*_∞_ [*m*/*s*]	log *ξ* [*g*/*mm*]	*t*_*e*_ [*ms*]
FFT	2.59	36	1745	-1.143	0.21
MST	19.1	36	1745	-0.275	0.21
SWT	53.5	36	1745	0.172	0.21

**Fig 14:**
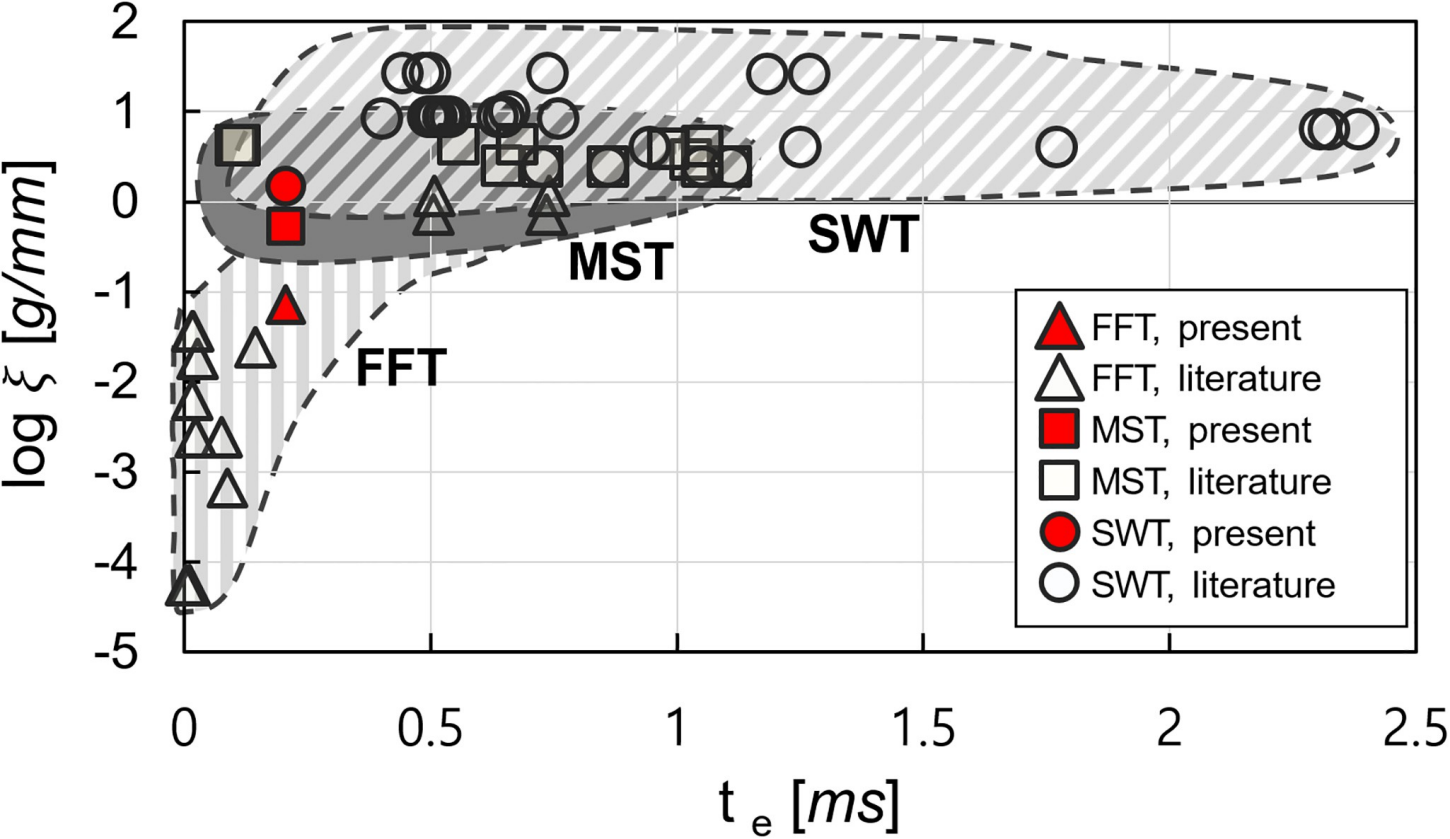
Range of size factor and flow establishment time for each technique. The FFT data obtained from Refs. [6, 10, 11, 48–50]; the MST data obtained from Refs. [7, 12, 13, 22, 24, 51, 52]; the SWT data obtained from Refs. [8, 9, 15, 22, 43–45, 53].

Fig 14 presents the range of the size factor *ξ* in log scale and the flow establishment time *t*_*e*_ of the FFT, MST, and SWT in literature and the present work. The shaded area in the figure represents the estimated range of the size factor and the flow establishment time for the FFT (patterned with a vertical line), MST (shaded without a pattern), and SWT (patterned with a diagonal line). The FFT, MST, and SWT data of the present work are included within the estimated range of each technique. It can be seen that the estimated range of the test environment are different between each technique.

Comparing each technique’s applied range of the size factor *ξ*, the FFT data was mainly distributed in the low size factor area, while the SWT data was distributed in a relatively high size factor area. And the MST was distributed between the size factor area of the FFT and SWT. For the flow establishment time *t*_*e*_ the acceleration measurement-based techniques, FFT and MST, were used for flow establishment times less than 1.1 *ms*, while the SWT, which is the strain gauge-based technique, was distributed in a broader range of the flow establishment times (from 0.1 to 2.4 *ms*). The difference in the applied range for each technique was mainly originated from the difference in the model-supporting mechanism of each technique.

For the FFT, the detachable supports are used, and the rigidity of the model-supporting mechanism is the weakest. Since the detachable wire can only hold a light test model, the logarithmic size factor log *ξ* for the FFT was very small, from -2.6 to 0.06 *g*/*mm*. In addition, the FFT was very susceptible to flow establishment. This is because the response time of the FFT was very short, and the unexpected motion of the free-flying model could be induced easily during the flow establishment time. For the FFT, one of the acceleration measurement-based techniques, the motion of the test model occurred immediately after the test flow reached the test model, which indicates that the response time of the drag measurement technique was very short. Since the test model was moving even during the flow establishment time around the test model, an unexpected motion of the test model could occur and induced a significant error in drag measurement. Therefore, the FFT had the shortest flow establishment time *t*_*e*_. If the test models are too heavy and large, the displacements of the free-flying model can be significantly small and the enormous errors can be induced for the acquisition of the test model’s nucleation from the shadowgraph images. For the heavy and large test model, internal mounting accelerometers in the test model [54] or improved image-based optical tracking [55, 56] could be applied to measure the accurate acceleration of the free-flying model.

Compared to the FFT, the MST had larger size factor due to the movable support, which has higher rigidity than the detachable support. Because of the movable support, the MST could hold a more heavier test model. The logarithmic size factor log *ξ* for the MST was from -0.27 to 0.65 *g*/*mm*, which means that the test model in the MST was dense. For the flow establishment time, the MST and the FFT had similar range. Because the MST is also an acceleration measurement-based techniques, similar to the FFT, the MST was used for the short flow establishment time less than 1.1 *ms* to minimize the unexpected motion during the flow establishment time.

For the SWT, the logarithmic size factor was from 0.17 to 1.43 *g*/*mm*, which indicates that heavy test models could be used. Since the strain gauge-based technique SWT obtained the drag from the structural deformation of the test model-support system, it was not necessary to decrease the rigidity of the model-supporting mechanisms to occur the movement of the test model; therefore, the SWT could use rigid supports to hold the test model. For the SWT’s flow establishment time *t*_*e*_, the applied range was from 0.2 to 2.5 *ms*. This is because the deformation of the system had a slow response time compared to the test model’s acceleration and could hold the test model with high-rigid support. Therefore, the SWT could minimize the unexpected motion generation and its effect on the drag measurement during the flow establishment time. Consequently, the SWT had the biggest size factor and the broadest range of the flow establishment time.

In the comparison, it can be seen that the difference in the size factor and the flow establishment time between drag measurement techniques was mainly originated from the rigidity of the model-supporting mechanism. The application of the FFT was strongly restricted by the test environments because the model-supporting mechanism of the FFT is very weak. Compared to the FFT, the MST could be used for the relatively heavy test model using the movable supports under a similar flow establishment time of the FFT. The SWT could use the model supports with high rigidity and could be used for the various test environments.

## Conclusions

Drag measurements in the shock tunnel were performed using three different drag measurement techniques; the FFT, the MST, and the SWT. For the use of each technique, the system setup, data processing method, and measurement uncertainties should be considered, which were described in detail for each technique. At the flow condition of freestream Mach number of 6, the obtained drag coefficients of the circular pointed cone model with a semi-angle of 18.4° using the FFT, MST, and SWT were 0.284, 0.301, and 0.265, respectively. The deviations of the experimental measurements from the numerical simulation were within 6.64%.

From an uncertainty analysis, it is observed that the total measurement uncertainty was decreased in the order of the FFT, SWT, and MST. The proportions of the Mach number, drag, nozzle-exit pitot pressure measurements to the total measurement uncertainty were examined. The uncertainty of Mach number measurement was the most significant. The uncertainties of the drag measurement techniques were mainly determined by the model-supporting mechanism and the signal processing for the drag coefficient acquisition. For the FFT, the measurement uncertainties were mainly induced from the corner detection uncertainties of the free-flying model. The MST had the smallest uncertainties because of the free-end configuration and the dynamic calibration, which minimize and calibrate the propagation of the external and internal vibrations. The SWT’s uncertainties were mainly originated from the propagation of the external vibration. Bending strain compensation system and vibration isolation system can be considered to reduce the SWT uncertainty.

The appropriate ranges of the test model size and flow establishment time for the application of the drag measurement technique were discussed with the comparison of the drag measurement results from the present work and the available literature. The appropriate range of the test environments for each technique was mainly determined by the model-supporting mechanism. The FFT had the estimated range with a low size factor and short flow establishment time, while the SWT was used for the broadest range of the size factor and the flow establishment time. The range of the MST had a similar flow establishment time to the FFT and had slightly larger size factors than that of the FFT. As a result, it can be seen that the FFT is appropriate for the drag measurement of the light and small test model with relatively simple signal processing. The MST can be used for the drag measurements of the middle size test model with relatively low measurement uncertainty. Using the SWT with a high accuracy dynamic calibration, which can use the rigid supports, the drag measurement of the heavy and larger test models can be performed.

## Supporting information

S1 Fig Fast response force measurement techniques for a short duration. (TIF)

S2 Fig Photographs of test model. (a) FFT, (b) MST, (c) SWT.
(TIF)

S3 Fig Mesh grid for numerical prediction of drag force. (a) FFT, (b) MST, (c) SWT.
(TIF)

S4 Fig Comparison between the calculation result and schlieren image for the MST. (TIF)

S1 Table Test model size and flow condition obtained from the available literature. (PDF)
