# Supplementary figures and images for: Assessment of drag measurement techniques in a shock tunnel
Source: PLoS One. 2022 Jul 8;17(7):e0270743. doi: 10.1371/journal.pone.0270743 (PMC9269454; doi:10.1371/journal.pone.0270743)

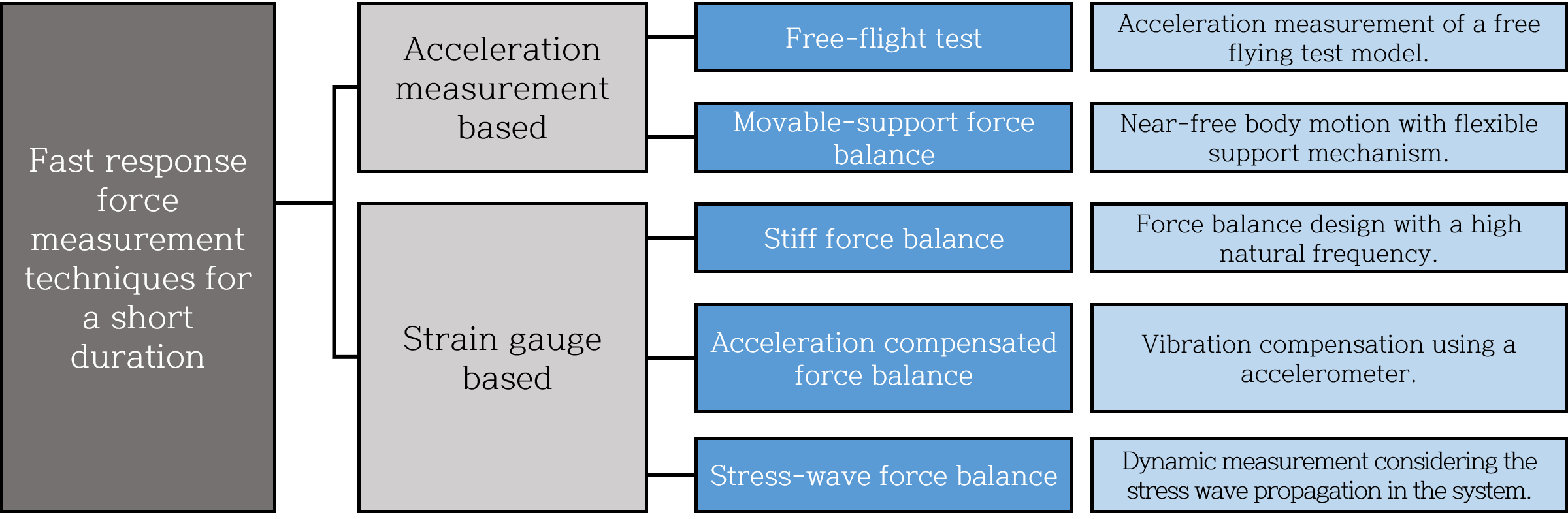

Supplement: S1 Fig — (TIF) [file pone.0270743.s001.tif]

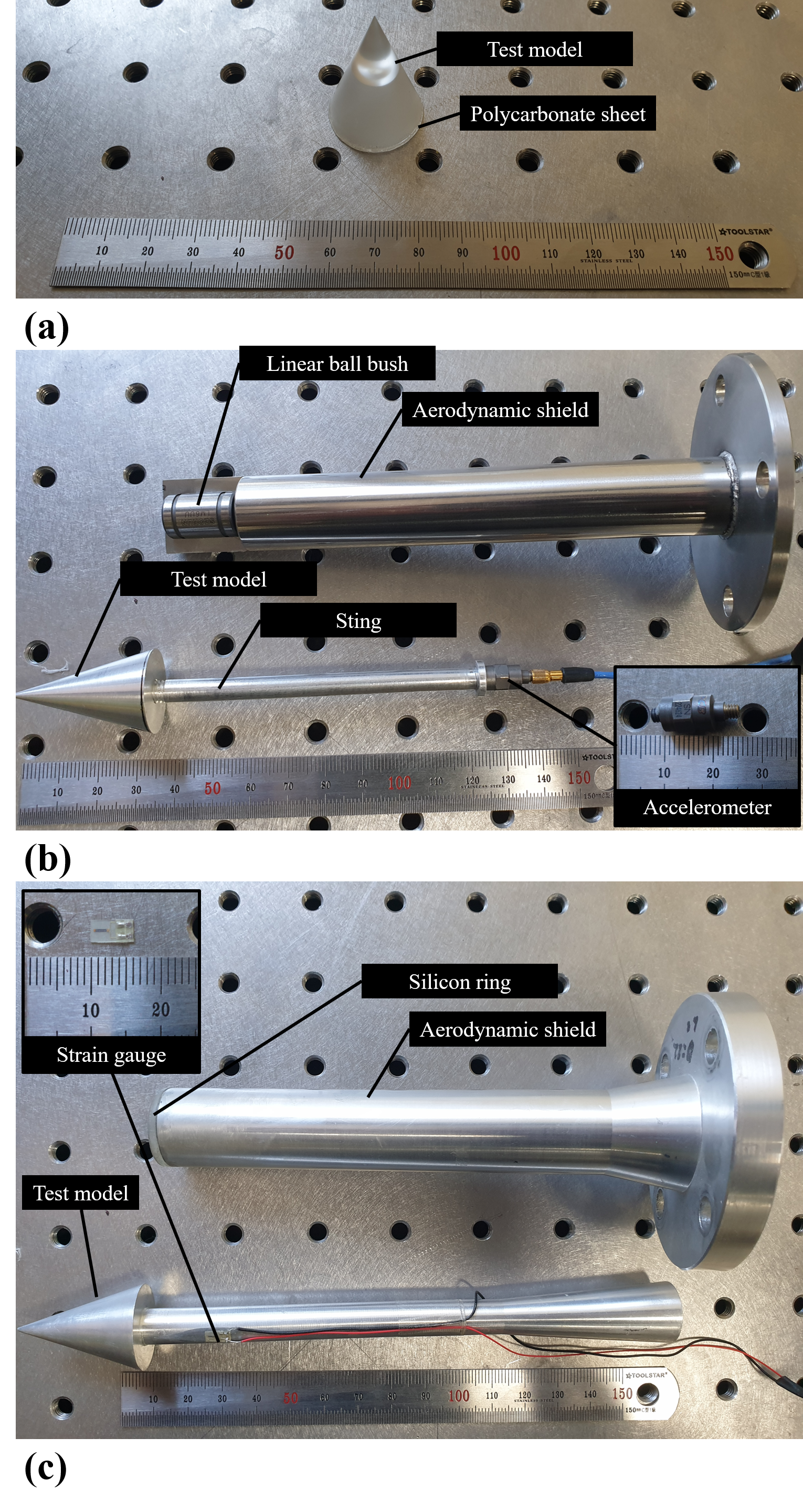

Supplement: S2 Fig — (a) FFT, (b) MST, (c) SWT. (TIF) [file pone.0270743.s002.tif]

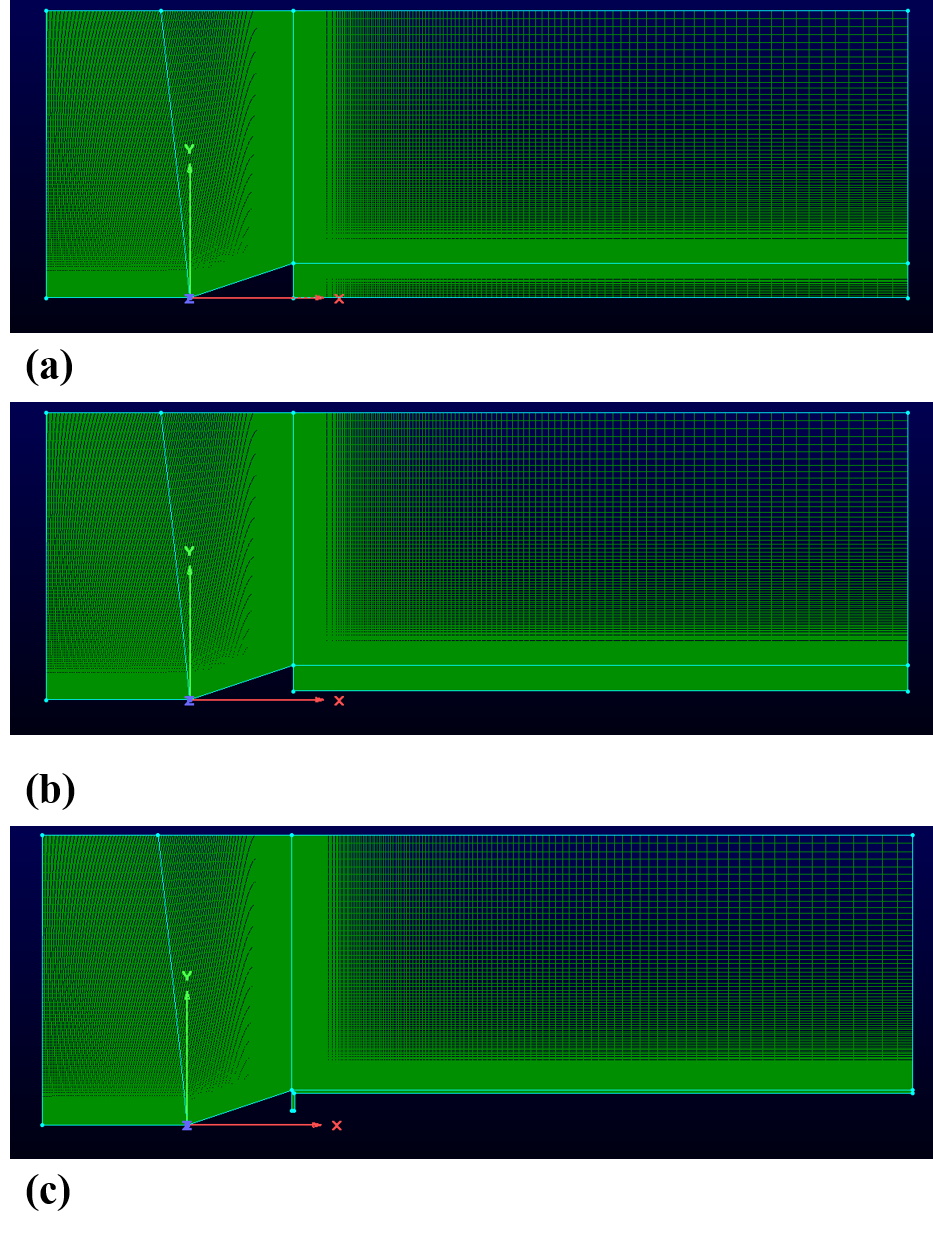

Supplement: S3 Fig — (a) FFT, (b) MST, (c) SWT. (TIF) [file pone.0270743.s003.tif]

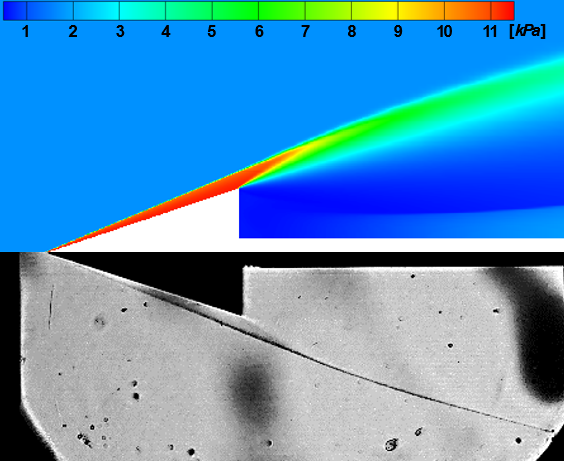

Supplement: S4 Fig — (TIF) [file pone.0270743.s004.tif]
