# Supplementary material for: Assessment of drag measurement techniques in a shock tunnel
Source: PLoS One. 2022 Jul 8;17(7):e0270743. doi: 10.1371/journal.pone.0270743 (PMC9269454; doi:10.1371/journal.pone.0270743)
Supplement: S1 Table — (PDF) [file pone.0270743.s005.pdf]

S1 Table 1. Test model dimension and flow condition obtained from the available literature.

Tech.	m $[g]$	l $[mm]$	v_{∞} $[m/s]$	t_e $[ms]$	$\log \xi$ $[g/mm]$	Ref.
FFT	2.59	36	1745	0.21	-1.14	Present work
	(0.9)	37.5	2588	0.14	-1.62	[6]
	0.11	3	(1758)	0.02	-1.44	[10]
	0.019	3	1870	0.02	-2.20	[11]
	0.089	5	1870	0.03	-1.75	
	0.11	3	1870	0.02	-1.44	
	(0.005)	2	262	0.08	-2.61	[48]
	(0.005)	2	857	0.02	-2.61	
	(0.00002)	0.3	262	0.01	-4.26	
	(0.00002)	0.3	857	0.004	-4.26	
	55.9	(76.82)	(1045)	0.74	-0.14	[49]
	55.9	(76.82)	(1522)	0.50	-0.14	
	90.3	(76.82)	(1045)	0.74	0.07	
	90.3	(76.82)	(1522)	0.51	0.07	
	(85.82)	50	2046	0.24	0.23	[50]
	(47.72)	60	2046	0.29	-0.10	
MST	19.1	36	1745	0.21	-0.28	Present work
	176	70.56	(1093)	0.65	0.40	[7]
	(340)	115	1120	1.03	0.47	[12]
	517	125	(1189)	1.05	0.62	[13]
	301	(121.24)	(1093)	1.11	0.39	[22]
	301	(121.24)	(1153)	1.05	0.39	
	301	(121.24)	(1412)	0.86	0.39	
	301	(121.24)	(1670)	0.73	0.39	
	274	65	5811	0.11	0.62	[24]
	274	65	5795	0.11	0.62	
	274	65	6190	0.11	0.62	
	274	65	5877	0.11	0.62	
	330.7	75.7	1360	0.56	0.64	[51]
	330.7	75.7	1120	0.68	0.64	
	480.9	125	(1273)	0.98	0.59	[52]
SWT	53.5	36	1745	0.21	0.17	Present work
	1700	200	5000	0.40	0.93	[8]
	1700	200	2640	0.76	0.93	
	1940	220	3390	0.65	0.95	[9]
	1940	220	3460	0.64	0.95	
	1940	220	4370	0.50	0.95	
	1940	220	4310	0.51	0.95	
	1940	220	4390	0.50	0.95	
	1940	220	4450	0.49	0.95	
	1940	220	4410	0.50	0.95	
	1940	220	4080	0.54	0.95	
	1940	220	4180	0.53	0.95	
	1940	220	4160	0.53	0.95	
	4940	182	2470	0.74	1.43	[15]
	4940	182	3760	0.48	1.43	
	4940	182	4110	0.44	1.43	
	4940	182	350	0.50	1.43	
	301	(121.24)	(1093)	1.11	0.39	[22]
	301	(121.24)	(1153)	1.05	0.39	
	301	(121.24)	(1412)	0.86	0.39	
	301	(121.24)	(1670)	0.73	0.39	
	1710	425	2400	1.77	0.60	[43]
	1710	425	3400	1.25	0.60	
	1710	425	4500	0.94	0.60	
	3600	567	2460	2.30	0.80	[44]
	3600	567	2440	2.32	0.80	
	3600	567	2380	2.38	0.80	
	8000	303	2390	1.27	1.42	[45]
	8000	303	2560	1.18	1.42	
	907	88.9	1350	0.66	1.01	[53]

* Numbers in parentheses indicate the calculated value from other available information.

* The test model mass is calculated using the model geometry and density in literature.

* The freestream velocity is calculated using the freestream Mach number and temperature in literature.
